# Supplementary material for: Maternal obesity and prenatal alcohol exposure are associated with child development: Results from the Safe Passage Study
Source: PLoS One. 2026 Apr 6;21(4):e0345406. doi: 10.1371/journal.pone.0345406 (PMC13052907; doi:10.1371/journal.pone.0345406)
Supplement: S1 Table — (DOCX) [file pone.0345406.s001.docx]

**S1 Table:** Amount smoking and drinking in the cluster groups by trimester

| **Exposure category** | **Exposure Information** | | |
| --- | --- | --- | --- |
|  | **Trimester 1** | **Trimester 2** | **Trimester 3** |
| ***Smoking category* Average Cigarettes/ week, mean (SD)** | | | |
| Non-smokers | 0.014 (0.08) | 0.003 (0.04) | 0.009 (0.06) |
| Quit early | 8.81 (9.92) | 0.07 (0.19) | 0.11 (0.22) |
| Low continuous | 15.72 (10.28) | 15.40 (10.79) | 14.33 (10.32) |
| Moderate to high continuous | 48.31 (21.71) | 51.71 (25.53) | 47.09 (25.29) |
| ***Drinking category* Drinks/ trimester, mean (SD)** | | | |
| Non-drinkers | 0.04 (0.16) | 0.02 (0.10) | 0.01 (0.07) |
| Quit early | 8.77 (7.41) | 0.12 (0.58) | 0.04 (0.26) |
| Low continuous | 2.41 (3.83) | 2.27 (2.71) | 1.85 (1.25) |
| Moderate to high continuous | 40.89 (60.08) | 16.12 (28.27) | 7.46 (16.88) |
