# Supplementary material for: Maternal obesity and prenatal alcohol exposure are associated with child development: Results from the Safe Passage Study
Source: PLoS One. 2026 Apr 6;21(4):e0345406. doi: 10.1371/journal.pone.0345406 (PMC13052907; doi:10.1371/journal.pone.0345406)
Supplement: S2 Table — (DOCX) [file pone.0345406.s002.docx]

**S2 Table:** Distribution of binge events by cluster group (Non-drinkers/Quit early/Low continuous/Moderate to high continuous), by site (Northern Plains, NP, Cape Town, CT), and trimester (T1/T2/T3)

|  | Non-drinkers | | Quit early | | Low continuous | | Moderate to high continuous | |
| --- | --- | --- | --- | --- | --- | --- | --- | --- |
|  | CT | NP | CT | NP | CT | NP | CT | NP |
|  | N = 777 | N = 552 | N = 325 | N =63 | N = 368 | N = 479 | N = 220 | N = 20 |
| **Binge events in Trimester 1** | | | | | | | | |
| 0 | 777 (100%) | 552 (100%) | 128 (39%) | 6 (9.5%) | 160 (43%) | 245 (51%) | 190 (86%) | 16 (80%) |
| 1 | 0 (0%) | 0 (0%) | 52 (16%) | 5 (7.9%) | 143 (39%) | 156 (33%) | 30 (14%) | 4 (20%) |
| 2 | 0 (0%) | 0 (0%) | 27 (8.3%) | 5 (7.9%) | 48 (13%) | 56 (12%) | 0 (0%) | 0 (0%) |
| ≥3 | 0 (0%) | 0 (0%) | 118 (36%) | 47 (75%) | 17 (4.6%) | 22 (4.6%) | 0 (0%) | 0 (0%) |
| **Binge events in Trimester 2** | | | | | | | | |
| 0 | 777 (100%) | 552 (100%) | 126 (39%) | 61 (97%) | 368 (100%) | 479 (100%) | 164 (75%) | 18 (90%) |
| 1 | 0 (0%) | 0 (0%) | 72 (22%) | 0 (0%) | 0 (0%) | 0 (0%) | 56 (25%) | 2 (10%) |
| 2 | 0 (0%) | 0 (0%) | 51 (16%) | 1 (1.6%) | 0 (0%) | 0 (0%) | 0 (0%) | 0 (0%) |
| ≥3 | 0 (0%) | 0 (0%) | 76 (23%) | 1 (1.6%) | 0 (0%) | 0 (0%) | 0 (0%) | 0 (0%) |
| **Binge events in Trimester 3** | | | | | | | | |
| 0 | 777 (100%) | 552 (100%) | 213 (66%) | 62 (98%) | 368 (100%) | 479 (100%) | 220 (100%) | 20 (100%) |
| 1 | 0 (0%) | 0 (0%) | 71 (22%) | 1 (1.6%) | 0 (0%) | 0 (0%) | 0 (0%) | 0 (0%) |
| 2 | 0 (0%) | 0 (0%) | 14 (4.3%) | 0 (0%) | 0 (0%) | 0 (0%) | 0 (0%) | 0 (0%) |
| ≥3 | 0 (0%) | 0 (0%) | 27 (8.3%) | 0 (0%) | 0 (0%) | 0 (0%) | 0 (0%) | 0 (0%) |
