# Supplementary figures and images for: Maternal obesity and prenatal alcohol exposure are associated with child development: Results from the Safe Passage Study
Source: PLoS One. 2026 Apr 6;21(4):e0345406. doi: 10.1371/journal.pone.0345406 (PMC13052907; doi:10.1371/journal.pone.0345406)

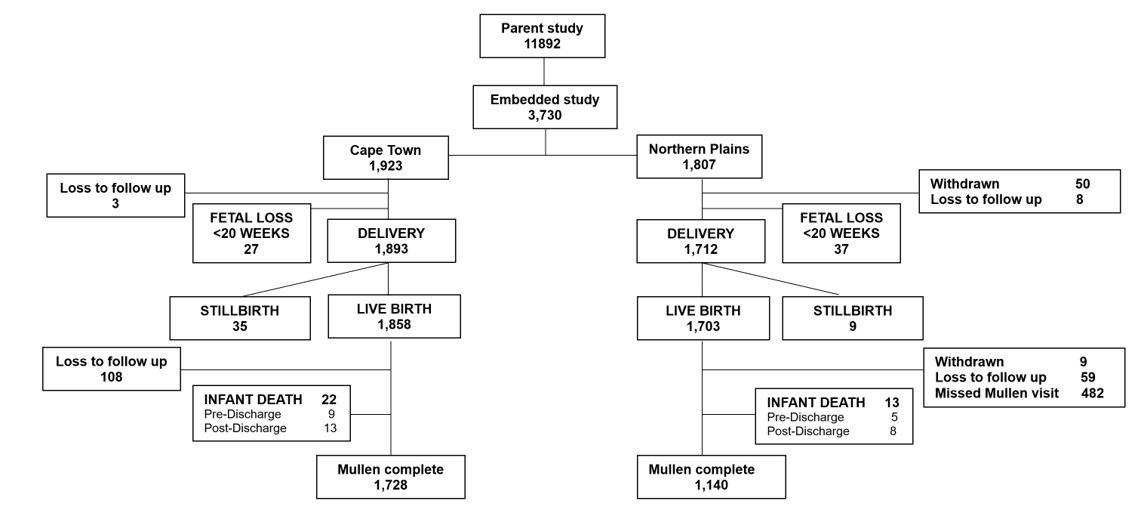

Supplement: S1 Fig — (JPG) [file pone.0345406.s003.jpg]
