# Supplementary material for: Maternal obesity and prenatal alcohol exposure are associated with child development: Results from the Safe Passage Study
Source: PLoS One. 2026 Apr 6;21(4):e0345406. doi: 10.1371/journal.pone.0345406 (PMC13052907; doi:10.1371/journal.pone.0345406)
Supplement: Attachment — Submitted filename: Maternal obesity and alcohol_Safe Passage_typos.pdf [file pone.0345406.s004.pdf]

PLOS ONE

Maternal obesity and prenatal alcohol exposure are associated with child development: Results from the Safe Passage Study

--Manuscript Draft--

**Manuscript Number:**	PONE-D-25-11614
**Article Type:**	Research Article
**Full Title:**	Maternal obesity and prenatal alcohol exposure are associated with child development: Results from the Safe Passage Study
**Short Title:**	Prenatal obesity, alcohol exposure and child development
**Corresponding Author:**	Ayesha Sania, Sc.D<br>Columbia University Department of Psychiatry<br>New York, New York UNITED STATES OF AMERICA
**Keywords:**	Child development, Infant Development, Prenatal alcohol use, Prenatal tobacco use, Mullen Scale of Early Development, Maternal obesity, Maternal depression
**Abstract:**	A large body of evidence supports the role of the prenatal environment in shaping childhood development. The relative contributions of prenatal alcohol use (PAE), maternal socioeconomic, and nutritional status on child development vary in high- versus low-income settings. We analyzed data from a prospective cohort study among mother-infant dyads from Northern Plains (NP), USA and Cape Town (CT), South Africa. The Mullen Scales of Early Learning were administered by trained assessors to evaluate cognitive, motor, and language development of 1-year old children. We used multiple linear regression models to assess standardized mean differences in development scores by prenatal and delivery factors within each study site. 1,140 infants from NP and 1,728 infants from CT were included in the analyses. In CT, infants with moderate-to-high PAE had 0.17 SD (95% CI -0.30, -0.04) lower cognitive and 0.15 SD (-0.29, -0.2) lower expressive language scores compared to infants without PAE. In the NP, maternal obesity (BMI>30 kg/m2) was significantly associated with -0.21 SD (-0.36, -0.06), and -0.13 SD (-0.27, -0.02) reductions in cognitive, and expressive language scores, respectively. Household crowding, lower levels of maternal educational attainment, prenatal maternal depression, low birthweight, admission to neonatal intensive care unit, and male sex had significant negative associations with cognitive and language development in both sites with effects ranging from -0.32 to -0.11 SDs. These results highlight the importance of assessing risk factors by populations across diverse social and cultural environments and emphasize the imperative to formulate intervention packages tailored to the local context.
**Order of Authors:**	Ayesha Sania, Sc.D
	Shreya Rao
	Nicolò Pini
	Mandy Potter
	Yael Rayport
	Liana Eisler
	Lucy Brink
	Jyoti Angal
	Michael M Myers
	Hein Odendaal
	Amy J Elliot
	William P Fifer
	Lauren C Shuffrey

Powered by Editorial Manager® and ProduXion Manager® from Aries Systems Corporation

Additional Information:	
**Question**	**Response**
**Financial Disclosure**<br><br>Enter a financial disclosure statement that describes the sources of funding for the work included in this submission. Review the submission guidelines for detailed requirements. View published research articles from *PLOS ONE* for specific examples.<br><br>This statement is required for submission and **will appear in the published article** if the submission is accepted. Please make sure it is accurate.<br><br>**Funded studies**<br>Enter a statement with the following details:<br>• Initials of the authors who received each award<br>• Grant numbers awarded to each author<br>• The full name of each funder<br>• URL of each funder website<br>• Did the sponsors or funders play any role in the study design, data collection and analysis, decision to publish, or preparation of the manuscript?<br><br>Did you receive funding for this work?	Yes
Please add funding details.<br>as follow-up to "**Financial Disclosure**<br><br>Enter a financial disclosure statement that describes the sources of funding for the work included in this submission. Review the submission guidelines for detailed requirements. View published research articles from *PLOS ONE* for specific examples.<br><br>This statement is required for submission and **will appear in the published article** if the submission is accepted. Please make sure it is accurate.	This research was supported by grants UH3OD023279, U01HD055154, U01HD045935, U01HD055155, and U01AA016501, issued by the Office of the Director of the National Institutes of Health, National Institute on Alcohol Abuse and Alcoholism, Eunice Kennedy Shriver National Institute of Child Health and Human Development, and the National Institute on Deafness and Other Communication Disorders. Ayesha Sania was supported by a career development award from the Fogarty International Center at the NIH (1K01TW012425-01A1). The opinions expressed in this paper are those of the authors and do not necessarily represent the official views of the National Institutes of Health, the Eunice Kennedy Shriver National Institute of Child Health and Development, or Fogarty International Center, the National Institute on Alcohol Abuse and Alcoholism, or the National Institute on Deafness and Other Communication Disorders.

**Funded studies**<br>Enter a statement with the following details:<br>• Initials of the authors who received each award<br>• Grant numbers awarded to each author<br>• The full name of each funder<br>• URL of each funder website<br>• Did the sponsors or funders play any role in the study design, data collection and analysis, decision to publish, or preparation of the manuscript?<br><br>Did you receive funding for this work?"	
Please select the country of your main research funder (please select carefully as in some cases this is used in fee calculation).<br>as follow-up to "**Financial Disclosure**<br><br>Enter a financial disclosure statement that describes the sources of funding for the work included in this submission. Review the submission guidelines for detailed requirements. View published research articles from *PLOS ONE* for specific examples.<br><br>This statement is required for submission and **will appear in the published article** if the submission is accepted. Please make sure it is accurate.<br><br>**Funded studies**<br>Enter a statement with the following details:<br>• Initials of the authors who received each award<br>• Grant numbers awarded to each author<br>• The full name of each funder<br>• URL of each funder website<br>• Did the sponsors or funders play any role in the study design, data collection and analysis, decision to publish, or preparation of the manuscript?<br><br>Did you receive funding for this work?"	UNITED STATES - US
**Competing Interests**	The authors have declared that no competing interests exist.

Use the instructions below to enter a competing interest statement for this submission. On behalf of all authors, disclose any competing interests that could be perceived to bias this work—acknowledging all financial support and any other relevant financial or non-financial competing interests.<br><br>This statement is required for submission and **will appear in the published article** if the submission is accepted. Please make sure it is accurate and that any funding sources listed in your Funding Information later in the submission form are also declared in your Financial Disclosure statement.<br><br>View published research articles from *PLOS ONE* for specific examples.<br><br>**NO authors have competing interests**<br><br>Enter: *The authors have declared that no competing interests exist.*<br><br>**Authors with competing interests**<br><br>Enter competing interest details beginning with this statement:<br><br>*I have read the journal's policy and the authors of this manuscript have the following competing interests: [insert competing interests here]*<br><br>* typeset	
**Ethics Statement**<br><br>Enter an ethics statement for this submission. This statement is required if the study involved:<br><br>• Human participants<br>• Human specimens or tissue<br>• Vertebrate animals or cephalopods<br>• Vertebrate embryos or tissues	The research was approved by the Institutional Review Board (IRB) of the National Institutes of Health (NIH). The study was conducted in accordance with ethical standards and received the necessary institutional approval for the use of human participants. Written consent was obtained for participation, with the assurance that the data were analyzed anonymously to protect participant privacy.

Powered by Editorial Manager® and ProduXion Manager® from Aries Systems Corporation

• Field research<br><br>Write "N/A" if the submission does not require an ethics statement.<br><br>General guidance is provided below. Consult the submission guidelines for detailed instructions. **Make sure that all information entered here is included in the Methods section of the manuscript.**	

**Format for specific study types**<br><br>**Human Subject Research (involving human participants and/or tissue)**<br>• Give the name of the institutional review board or ethics committee that approved the study<br>• Include the approval number and/or a statement indicating approval of this research<br>• Indicate the form of consent obtained (written/oral) or the reason that consent was not obtained (e.g. the data were analyzed anonymously)<br><br>**Animal Research (involving vertebrate animals, embryos or tissues)**<br>• Provide the name of the Institutional Animal Care and Use Committee (IACUC) or other relevant ethics board that reviewed the study protocol, and indicate whether they approved this research or granted a formal waiver of ethical approval<br>• Include an approval number if one was obtained<br>• If the study involved *non-human primates*, add *additional details* about animal welfare and steps taken to ameliorate suffering<br>• If anesthesia, euthanasia, or any kind of animal sacrifice is part of the study, include briefly which substances and/or methods were applied<br><br>**Field Research**<br><br>Include the following details if this study involves the collection of plant, animal, or other materials from a natural setting:<br>• Field permit number<br>• Name of the institution or relevant body that granted permission	
**Data Availability**<br><br>Authors are required to make all data underlying the findings described fully available, without restriction, and from the time of publication. PLOS allows rare exceptions to address legal and ethical concerns. See the PLOS Data Policy and FAQ for detailed information.	No - some restrictions will apply

A Data Availability Statement describing where the data can be found is required at submission. Your answers to this question constitute the Data Availability Statement and **will be published in the article**, if accepted.<br><br>**Important:** Stating 'data available on request from the author' is not sufficient. If your data are only available upon request, select 'No' for the first question and explain your exceptional situation in the text box.<br><br>Do the authors confirm that all data underlying the findings described in their manuscript are fully available without restriction?	
**Describe where the data may be found in full sentences. If you are copying our sample text, replace any instances of XXX with the appropriate details.**<br><br>• If the data are **held or will be held in a public repository**, include URLs, accession numbers or DOIs. If this information will only be available after acceptance, indicate this by ticking the box below. For example: *All XXX files are available from the XXX database (accession number(s) XXX, XXX.*).<br>• If the data are all contained **within the manuscript and/or Supporting Information files**, enter the following: *All relevant data are within the manuscript and its Supporting Information files.*<br>• If neither of these applies but you are able to provide **details of access elsewhere**, with or without limitations, please do so. For example:<br><br>*Data cannot be shared publicly because of [XXX]. Data are available from the XXX Institutional Data Access / Ethics Committee (contact via XXX) for researchers who meet the criteria for access to confidential data.*<br><br>*The data underlying the results presented in the study are available from (include the name of the third party*	Data cannot be shared publicly because of ethical concerns regarding the use of data contributed by historically marginalized populations. Data are available from the Avera Research Institute and Stellenbosch University Institutional Data Access (contact via Amy Elliott, Amy.Elliott@avera.org and Hein Odendaal, hjo@sun.ac.za) for researchers who meet the criteria for access to confidential data.

and contact information or URL).<br>• This text is appropriate if the data are owned by a third party and authors do not have permission to share the data.<br><br>* typeset	
Additional data availability information:	

Manuscript
Click here to access/download;Manuscript;Mullen Manuscript 03252025.docx

Maternal obesity and prenatal alcohol exposure are associated with child development: Results from the Safe Passage Study

Ayesha Sania[1], Shreya Rao[1], Nicolò Pini[1], Mandy Potter[2], Yael Rayport[1], Liana Eisler[1], Lucy Brink[2], Jyoti Angal[3,4], Michael M. Myers[1], Hein Odendaal[2], Amy J. Elliott[3,4], William P. Fifer*[1,5,6], Lauren C. Shuffrey*[7]

*Denotes shared senior authorship

1. Department of Psychiatry, Columbia University Irving Medical Center, New York, NY, 10032, USA.
2. Department of Obstetrics and Gynaecology, Faculty of Medicine and Health Science, Stellenbosch University, Cape Town, Western Cape, South Africa 7530
3. Avera Research Institute, Sioux Falls, SD, USA.
4. Department of Pediatrics, University of South Dakota Sanford School of Medicine, Sioux Falls, SD, USA
5. Division of Developmental Neuroscience, New York State Psychiatric Institute, New York, NY, 10032, USA.
6. Department of Pediatrics, Columbia University Irving Medical Center, New York, NY, 10032, USA.
7. Department of Child and Adolescent Psychiatry, NYU Grossman School of Medicine, New York, NY, 10016, USA.

Funding information:

1

Abstract:

A large body of evidence supports the role of the prenatal environment in shaping childhood development. The relative contributions of prenatal alcohol use (PAE), maternal socioeconomic, and nutritional status on child development vary in high- versus low-income settings.

We analyzed data from a prospective cohort study among mother-infant dyads from Northern Plains (NP), USA and Cape Town (CT), South Africa. The Mullen Scales of Early Learning were administered by trained assessors to evaluate cognitive, motor, and language development of 1-year old children. We used multiple linear regression models to assess standardized mean differences in development scores by prenatal and delivery factors within each study site.

1,140 infants from NP and 1,728 infants from CT were included in the analyses. In CT, infants with moderate-to-high PAE had 0.17 SD (95% CI -0.30, -0.04) lower cognitive and 0.15 SD (-0.29, -0.2) lower expressive language scores compared to infants without PAE. In the NP, maternal obesity (BMI>30 kg/m^2) was significantly associated with -0.21 SD (-0.36, -0.06), and -0.13 SD (-0.27, -0.02) reductions in cognitive, and expressive language scores, respectively. Household crowding, lower levels of maternal educational attainment, prenatal maternal depression, low birthweight, admission to neonatal intensive care unit, and male sex had significant negative associations with cognitive and language development in both sites with effects ranging from -0.32 to -0.11 SDs.

These results highlight the importance of assessing risk factors by populations across diverse social and cultural environments and emphasize the imperative to formulate intervention packages tailored to the local context.

Introduction:

Understanding the predictors of early childhood development to support policy and intervention programs is a key priority worldwide, particularly in low-to-middle income countries (LMICs) where the majority of the world's children reside. According to recent estimates, a third (80 million) of the 3 and 4-year-old children in LMICs do not attain their full developmental potential (1). Early cognitive, language, and socioemotional development are key determinants of future educational and income achievement in adult life (2). Suboptimal development in the early childhood period can have a lasting impact on the life course of individuals as well as the larger society. Therefore, understanding the independent contributions prenatal exposures on child developmental outcomes is a significant global public health priority.

The prenatal period is a critical developmental window that has the potential to shape long-term health outcomes for offspring through fetal programming mechanisms. A growing body of research within the developmental origins of health and disease (DOHaD) framework highlights how adverse in-utero environmental factors including poor maternal nutritional status, exposure to harmful chemicals, crowded living conditions, and socioeconomic challenges impact children's cognitive, motor, and language development (3-6). The impact of risk factors on child development varies between high- and low-income settings, reflecting important contextual differences. For example, while studies in high-income contexts often report no effect or slight positive effects of prenatal alcohol exposure (PAE) on children's cognitive skills (7-9), research from low-income settings consistently reports harmful effects on both cognitive and motor skills (10-13). However, in studies from high-income countries, the apparent protective effect of mild drinking is potentially explained by the confounding effects of maternal education and favorable socioeconomic conditions (14-16). In addition, the null effects of PAE on child development in high income settings could be due to an enriched postnatal environment which acts as a buffer to the harmful effects of PAE on child development.

The role of prenatal maternal nutritional status (maternal anemia, short stature, and low body mass index [BMI] during pregnancy) (17, 18) on child development is well established. While studies from LMICs report association of low BMI during pregnancy with poor child development, recent studies report maternal obesity (BMI >30 kg/m2) as an emerging risk factor

for poor child health and development primarily in high-income settings (19-22). Recent systematic reviews of studies from high income countries report association of maternal obesity with lower cognitive development scores, developmental delay, attention deficit hyperactive disorder (ADHD, and autism spectrum disorder (ASD) (23, 24). Given the increasing rates of obesity worldwide, further studies are needed to examine this relationship in both high- and low-income settings.

In the present study, we estimated the associations of several prenatal exposures including PAE, maternal obesity, delivery and birth characteristics, and socioeconomic characteristics, with children's motor, cognitive, and language development in two large socioeconomically, ethnically, and culturally diverse cohorts of mother-infant dyads from the USA and South Africa using data from the NIH Safe Passage Study (25). Estimating the relative contributions of known and emerging risk factors, while considering contextual factors in both high-income and LMICs, can help inform programs and policies aimed at targeting interventions for at-risk infants.

Methods:

Study population

Participants were enrolled in the Safe Passage Study by the Prenatal Alcohol and SIDS and Stillbirth (PASS) network which was designed to examine the effects of prenatal drinking and smoking on adverse birth outcomes (25). This multi-site study included data from Western Cape Province, South Africa and five sites from the Northern Plains, United States, three of which were on American Indian Reservations. Women were eligible to participate in the study if they were 16 years or older, pregnant with one or two fetuses with a gestational age of 6 weeks or more, spoke English or Afrikaans, and were not planning to relocate from the catchment area during the study period. The study design has previously been described in detail by Dukes et al (26). Between August 2007 and January 2015, approximately 12,000 women enrolled in the study, of them a subset of 3750 women who enrolled prior to 24 weeks of gestation were randomly selected for additional study assessments during postnatal period. The analysis presented in this manuscript includes all singleton children with early childhood development assessment data from the Northern Plains (NP), USA and Cape Town (CT), South Africa.

Developmental assessment:

Child development was assessed with the Mullen Scales of Early Learning (MSEL) when the children were 1 year old. Infants born prematurely (<37 weeks gestation) were assessed at their adjusted postnatal age of 1-year. The Mullen is standardized play-based developmental assessment that assesses fine motor, gross motor, visual reception, expressive language, and receptive language function standardized to a T distribution. The cognitive T score in the MSEL is derived from the sum of visual reception, fine motor, receptive language, and expressive language subscales. The Mullen has been adapted to and validated in Afrikaans to South African context (27). The Mullen has also been used to assess the developmental trajectories of Native American children in the Northern Plains (28). In both locations, trained research staff administered the Mullen assessments.

Assessment of risk factors:

Socioeconomic data.

Information on marital/partner status was collected at the time of enrollment, and household crowding (ratio of number of people per room in a household) was collected at the first prenatal visit.

Maternal-Infant chart abstraction.

Data on maternal medical diagnoses, birth and delivery characteristics including gestational diabetes mellitus, hypertension, preeclampsia, induced or augmented labor, cesarean section, infant resuscitation, NICU admission, infant sex, and birthweight were collected from chart abstraction by research midwives.

Maternal anthropometrics.

Maternal height and weight were collected at the enrollment visit. The median gestational age at enrollment was 20 weeks (inter quartile range, IQR: 14, 25) in Cape Town and 16 weeks (IQR: 13, 18) in Northern Plains.

Alcohol and tobacco use during pregnancy.

Prenatal drinking and smoking information was collected longitudinally during up to four prenatal visits. A 30-day timeline follow-back interview was used to assess prenatal alcohol and tobacco exposure (29). During up to four prenatal visits, participants reported the day they most recently drank or smoked, and their substance use for the 30-days prior to it. They also described the quantity, sharing, and frequency of alcohol and cigarette consumption. The information was used to estimate the standard number of drinks of alcohol consumed per day of pregnancy and the average number of cigarettes smoked per week of pregnancy. Using clustering methods previously described, based on the amount and timing of drinking and smoking, the participants were divided into 4 smoking and 10 drinking groups (30). Because several of the drinking groups had a small number of subjects, they collapsed into 4 (non-smoker/drinker, quit early, low continuous, and moderate to high continuous) smoking and drinking groups for prior analyses (31, 32) and the current analyses.

Prenatal maternal depression assessment.

Maternal depression was assessed using the Edinburgh Postnatal Depression Scale (EPDS), which has been validated as a screening instrument in South Africa (33). An EPDS cutoff was used to categorize women into no depression (EPDS<10), probable depression (EPDS 10-13), and major depressive disorder (EPDS >13) groups.

Statistical analysis

To ensure comparability between the measurement in the two study sites, all development outcome scores were standardized (z-scored). Within each study site, linear regression models were used to assess standardized mean differences (SMDs) in cognitive, fine motor, gross motor, expressive language, and receptive language scores for the selected risk factors. Initially we fit a series of minimally adjusted models with each risk factor, child age and sex. We then included all variables with a p-value of 0.20 into the multivariable model.

We used separate models for three sets of risk factors based on their timing of measurement during the study (Figure S1). This allows us to avoid introduction of bias in our analyses by adjusting for variables on the hypothesized causal pathway between a risk factor and child development. The three models with different sets of predictors are: (1) model 1 included maternal socio-economic, demographic, drinking, smoking, and nutritional characteristics; (2) model 2 included pregnancy complications such as hypertensive disorders of pregnancy, gestational diabetes, and socio-economic indicators; (3) model 3 included delivery characteristics including location of delivery, birthweight, need for resuscitation and NICU admission and socio-economic indicators. All multivariable models included child's biological assigned sex at birth and age at assessment. Missing values for covariates were replaced with a "missing" indicator and included in the multivariable models. Because we have related outcomes and covariates, adjustment for multiple comparisons was not appropriate (34-36). All analyses were performed in SAS software version 9.4 (SAS Institute, Cary NC).

Ethical approval:

Written informed consent for the Safe Passage Study and a separate informed consent for the follow-up assessments were obtained. Ethical approval for the Safe Passage study was acquired

from the Health Research Ethics Committee of Stellenbosch University, Sanford Health, the Indian Health Service, participating Tribal Nations, and the New York State Psychiatric Institute. The data for this analysis were accessed between 24/06/2023 and 11/09/2024 in accordance with the approved data use agreements.

Results:

The final analyses included 2868 infants, of them 1140 (40%) were from the Northern Plains and 1728 (60%) were from Cape Town. Table 1 shows the characteristics of the study participants by site. Overall, participants from CT had poorer socioeconomic conditions, as reflected in their lower educational attainment, higher crowing index, less frequent access to antenatal care, and higher unemployment rates. Women in NP were taller, and a greater proportion were overweight and obese. In Cape Town, 23% women continued smoking in moderate to high amount throughout the pregnancy compared to only 4% woman in Northern Plains. Women in the moderate-to-high continuous group smoked 48.31 (SD 21.71) cigarettes/week, on average in first trimester. The majority (43%) of NP participants quit drinking in the first trimester, only 6% drank moderate-to-high amount, while 19% participants from CT continued to drink in moderate to high amount throughout pregnancy. Women in the high moderate-to-continuous group drank an average total drink of 40. 9 (SD 60.1) drinks in first trimester (Supplemental Table 1).

Predictors of child development in Cape Town:

The estimates of the association of predictors with infant development scores in Cape Town are presented in figures 1-5. PAE was associated with reduced cognitive and expressive language scores. Infants with moderate to high levels of PAE had 0.17 SD (95% Confidence Interval, CI: -0.30, -0.04) lower cognitive scores and 0.15 SD (CI -0.29, -0.02) lower expressive language scores, as compared with infants with no PAE. Indicators of SES such as maternal education and crowding index, markers of socioeconomic conditions, were associated with expressive language and gross motor scores. In comparison to infants of mothers with more than a high school education, infants of mothers with primary school education had 0.35 SD (CI: -0.64, -0.06) lower expressive language scores and 0.33 SD (CI: -0.2, -0.03) gross motor scores. Infants of mothers

with probable depression (EPDS 13-15) had lower gross motor scores (-0.17 SD, CI: -0.33, -0.02) compared to mothers without depression. With each unit increase in the crowding index, there was 0.07 SD and 0.06 SD reduction in cognitive and expressive language scores, respectively. We found no significant association of parity, number of antenatal care visits, maternal BMI during pregnancy, or marital/partner status with infant developmental outcomes on the MSEL.

Among the child and delivery factors, child sex, low birthweight, and admission to NICU were each independently associated with infant development on the MSEL. Compared to normal birthweight infants, infants born with birthweight below <2000 g had on average 0.46 SD (CI: -0.73, -0.020) lower cognitive and 0.55 SD (CI: -0.85, -0.25) lower fine motor scores. Infants who were admitted to the NICU after birth had 0.26 SD (CI: -0.45, -0.06), 0.28 SD (CI: -0.49, -0.07), and 0.31 SD (CI: -0.49, -0.12) lower cognitive expressive and receptive language scores, respectively. Female infants had on average higher cognitive, expressive, and receptive language scores compared to male infants. We did not find any significant associations between resuscitation at birth with any development domains on the MSEL.

Predictors of child development in Northern Plains:

The estimates of association of predictors with infant development in the Northern Plains site are shown in figures 6-10. We found significant associations of high maternal BMI during pregnancy with cognitive and language scores. Compared to infants of mother with normal early pregnancy BMI (18.5-25 kg/m^2), infants of obese mothers had on average 0.21 SD (CI: -0.37, -0.06) lower cognitive, 0.18 SD (CI -0.32, -0.03) lower receptive language, and 0.13 SD (CI: -0.28, 0.00) lower expressive language scores on the MSEL. Low BMI (<18.5 kg/m^2) was associated with 0.48 SD reduction in cognitive scores (CI: -0.90, -0.06). Among the indicators of socioeconomic status, crowding index was associated with cognitive and fine motor scores and maternal education was associated with fine motor scores. With each unit increase in the crowding index, there was 0.14 SD (CI: -0.26, -0.03) reduction in cognitive and a 0.10 SD (CI: -0.21, -0.02) reduction in fine motor scores. Infants of mothers with primary education had 0.97 SD (CI: -1.67, -0.26) lower fine motor scores compared to infants of mothers with more than

high school education. We did not find any significant association of prenatal drinking, smoking, or maternal depression with development scores.

Similar to the CT site, being born with low birthweight and having admission to the NICU were both independently linked with lower developmental scores in the NP. Compared to infants with normal birthweight, infants born with very to moderate low birthweight (<2000 g) and moderate low birthweight (2000-2500 g) had -0.62 SD and -0.33 SD lower cognitive scores, respectively. Infants who were born with very to moderate low birthweight (-0.74 SD, CI: -1.28, -0.21) and moderate birthweight (-0.38 SD, -0.72, -0.05) had lower gross motor scores compared to normal birthweight infants. Infants who were admitted to NICU had 0.29 SD (95% CI: -0.50, -0.09) lower cognitive, 0.22 SD (95% CI: -0.41, -0.03) lower receptive language scores and 0.33 SD (95% CI -0.54, -0.12) lower expressive language scores, compared to children who were not admitted to NICU. Across both sites, female infants had higher cognitive, and receptive and expressive language scores compared to male infants.

Discussion:

In this prospective cohort study among 2868 mother-infant dyads from the Northern Plains of the USA and Cape Town, South Africa, we identified associations of several prenatal maternal and birth factors with infant cognitive, language, and motor development at one year of age. We found a significant association of PAE with lower cognitive and language development of infants in Cape Town. Early pregnancy maternal obesity was associated with lower cognitive and language development scores in the Northern Plains. In addition, multiple risk factors classically associated with child development including lower levels of maternal educational attainment, crowded living conditions, being born with low birthweight, and NICU admission were significant predictors of lower neurodevelopmental scores in both study sites. We did not find significant associations of prenatal tobacco use and pregnancy complications with infant development outcomes in these analyses.

We observed adverse effects of moderate to high continuous PAE on cognitive and language development in the Cape Town site. These findings align closely with previous studies from LMICs that have reported associations between prenatal drinking and deficits in attention, learning, and memory, as well as social and emotional development (37-40). Similar to previous studies conducted in high income countries, we found no effects of PAE in the NP. One potential explanation is that the magnitude and duration of moderate-to-high-continuous PAE was much lower in the NP site (19% in CT compared to 4% in CT). This observation is supported by reports of dose-dependent effects of prenatal drinking, including a study in Danish population, which reported poor development among children whose mothers drank more than 9 drinks per week (41, 42). The observed null effect in the NP site could also be, in part, due to a favorable postnatal environment. We accounted for the same set of uniformly measured confounding variables in both study sites, therefore residual confounding is an unlikely explanation for the null effect in NP site. The differential impact of prenatal alcohol exposure (PAE) on development in South African infants, compared to American infants, can be due to the protective influence of adequate nutritional intake among populations in the United States, in contrast to the nutritional deficiencies prevalent in low-resource communities in South Africa (43, 44). Although alcohol is a teratogen that directly crosses the placenta into fetal blood

circulation, prior research has demonstrated dietary deficiencies, such as the essential nutrient choline, can exacerbate the teratogenic effects of PAE on offspring physical and behavioral development (45-47).

Our findings indicating that maternal obesity is associated with lower cognitive and language development in the NP site are strengthened by research conducted in high income settings (48). In addition to child cognition and language development, prior studies reported association of maternal obesity with increased risk for autism, ADHD, emotion regulation and behavioral problems in childhood (24, 49). Increased levels of prenatal maternal inflammation are a possible biological mechanism underlying these associations (50). There is limited data on the effects of maternal obesity on child development from LMIC settings, although rates of maternal obesity are rising in those contexts (51). Our observation of no association of maternal obesity in the Cape Town site could be due to lower rates of overweight and obesity (63% in NP compared to 42% in CT). In addition, BMI was measured later during pregnancy in the Cape Town site. BMI measured in later gestation, in part, reflects gestational weight gain. Thus, the null association in the CT site could also be explained by measurement error in the BMI assessment.

In our study, multiple indicators of socioeconomic status including maternal education and household crowding were associated with poor development in infants in both study sites. The unique contribution of our study is that we demonstrate detrimental effects of household crowding on multiple domains of child development across study sites. Chronic residential crowding has been linked to behavior problems and lower educational attainment in high income settings (52, 53). Analysis of the cross-sectional survey data from LMICs shows association of household crowding with poor cognitive and socioemotional development (54). The estimates of household crowding accounts for common correlates including parity and other indicators of SES, supporting that crowding impacts on infant development independently which is likely mediated via reduced maternal responsiveness in chaotic environments (55). Taken together, our findings indicate that household crowding, along with other easily assessable markers including maternal educational attainment, low birthweight and NICU admissions, can be used for targeting infants for developmental screening and interventions.

The strength of our study includes prospective data from a large sample of infants from two socioeconomically and ethnically diverse populations. We collected data on prenatal exposures, socioeconomic status, nutritional status, and other covariates using uniform data collection tools and assessment schedules which allowed comparability of the estimates across study sites. However, our study has several limitations including lack of data on postnatal environmental exposures such as postnatal maternal depression, early stimulation and responsive parenting practices which are major determinants of early childhood development (56). We used data from the PASS study which was conducted among populations with high prevalence of prenatal drinking and smoking, therefore the results of this study may not be directly generalizable to populations with a low prevalence of these exposures.

In summary, the contribution of our study is two-fold. First, we demonstrate the influence of risk factors classically associated with poor neurodevelopment is similar across study sites. Our findings indicate that household crowding, maternal educational attainment, low birthweight and NICU admission are easily assessable markers that can help identify infants in both high- and low-income settings for developmental screening and interventions. Second, our results support differential effects of PAE and maternal obesity across socioeconomic and cultural contexts. These findings highlight the importance of comprehensive intervention packages that are appropriate for the local context to be implemented for pregnant women and women of child-bearing age.

Table 1: Characteristics of the study population

**Characteristics**	**Cape Town, South Africa**	**Northern Plaines, USA**	**Overall**
	**(N=1728)**	**(N=1140)**	**(N=2868)**
**Maternal factors**			
**Maternal age (years)**			
Mean (SD)	25 (± 5.8)	27 (± 5.1)	26 (± 5.6)
**Height (cm)**			
<145	14 (1 %)	20 (2 %)	53 (2%)
145-150	119 (7 %)	7 (1 %)	126 (4 %)
150-155	348 (20 %)	35 (3 %)	383 (13 %)
>155	1228 (71 %)	1078 (95 %)	2306 (80 %)
Missing	19 (1.1%)		39 (1.4%)
**BMI (kg/m^2)**			
<18.5 underweight	133 (8 %)	8 (1 %)	141 (5 %)
18.5-25 normal	856 (50 %)	389 (34 %)	1245 (43 %)
25-30 overweight	361 (21 %)	334 (29 %)	695 (24 %)
>30 obese	355 (21 %)	386 (34 %)	741 (26 %)
Missing	23 (1 %)	23 (2 %)	46 (2 %)
**Parity**			
0	695 (40 %)	411 (36 %)	1106 (39 %)
1	532 (31 %)	344 (30 %)	876 (31 %)
2	323 (19 %)	217 (19 %)	540 (19 %)
3 or more	177 (10 %)	168 (15 %)	345 (12 %)
Missing	1 (0 %)	0 (0 %)	1 (0 %)
**Antenatal care visits**			
<3	122 (7 %)	7 (1 %)	129 (4 %)
3-6	966 (56 %)	53 (5 %)	1019 (36 %)
>6	592 (34 %)	1072 (94 %)	1664 (58 %)
Missing	48 (3 %)	8 (1 %)	56 (2 %)
**Education**			
Primary school education	122 (7 %)	12 (1 %)	134 (5 %)
Some high school education	1136 (66 %)	165 (14 %)	1301 (45 %)
High school completed	382 (22 %)	176 (15 %)	558 (19 %)
Higher than high school	85 (5 %)	787 (69 %)	872 (30 %)
Missing	3 (0 %)	0 (0 %)	3 (0 %)
**Marital status**			
Unmarried	885 (51 %)	229 (20 %)	1114 (39 %)

Married/Cohabiting	838 (48 %)	911 (80 %)	1749 (61 %)
Missing	5 (0 %)	0 (0 %)	5 (0 %)
**Employment status**			
Unemployed	985 (57 %)	270 (24 %)	1255 (44 %)
Employed	563 (33 %)	794 (70 %)	1357 (47 %)
Missing	180 (10 %)	76 (7 %)	256 (9 %)
**Crowding index (persons/room)**			
Mean (SD)	1.6 (± 0.86)	0.74 (± 0.68)	1.2 (± 0.89)
Missing	14 (0.8%)	16 (1.4%)	30 (1.0%)
**Maternal depression**			
None (EPS score <13)	846 (49 %)	1059 (93 %)	1905 (66 %)
Probable depression (EPS score 13-15)	216 (12 %)	34 (3 %)	250 (9 %)
Major depression (EPDS score >15)	666 (39 %)	47 (4 %)	713 (25 %)
**Prenatal drinking**			
None	785 (45 %)	565 (50 %)	1350 (47 %)
Moderate-high continuous	325 (19 %)	63 (6 %)	388 (14 %)
Low continuous	86 (5 %)	13 (1 %)	99 (3 %)
Quit early	369 (21 %)	485 (43 %)	854 (30 %)
Missing	163 (9.4%)	14 (1.2%)	177 (6.2%)
**Prenatal smoking**			
None	703 (41 %)	886 (78 %)	1589 (55 %)
Moderate-high continuous	392 (23 %)	48 (4 %)	440 (15 %)
Low continuous	573 (33 %)	124 (11 %)	697 (24 %)
Quit early	53 (3 %)	62 (5 %)	115 (4 %)
Missing	7 (0.4%)	20 (1.8%)	27 (0.9%)
**Pregnancy and delivery complications**			
**Gestational diabetes mellitus**			
No	1635 (95 %)	977 (86 %)	2612 (91 %)
Yes	12 (1 %)	76 (7 %)	88 (3 %)
Missing	81 (4.7%)	87 (7.6%)	168 (5.9%)
**Gestational hypertension**			
No	1604 (93 %)	1092 (96 %)	2696 (94 %)
Yes	69 (4 %)	41 (4 %)	110 (4 %)
Missing	55 (3.2%)	7 (0.6%)	62 (2.2%)
**Preeclamspia**			
No	1617 (94 %)	1091 (96 %)	2708 (94 %)
Yes	56 (3 %)	42 (4 %)	98 (3 %)
Missing	55 (3.2%)	7 (0.6%)	62 (2.2%)

**Induced Labor**			
No	1451 (84 %)	810 (71 %)	2261 (79 %)
Yes	205 (12 %)	315 (28 %)	520 (18 %)
Missing	72 (4.2%)	15 (1.3%)	87 (3.0%)
**Augmented labor**			
No	1472 (85 %)	796 (70 %)	2268 (79 %)
Yes	184 (11 %)	329 (29 %)	513 (18 %)
Missing	72 (4.2%)	15 (1.3%)	87 (3.0%)
**Cesarean section**			
No	1459 (84 %)	832 (73 %)	2291 (80 %)
Yes	261 (15 %)	289 (25 %)	550 (19 %)
Missing	8 (0.5%)	19 (1.7%)	27 (0.9%)
**Child factors**			
**Resuscitated at birth**			
No	1587 (92 %)	907 (80 %)	2494 (87 %)
Yes	135 (8 %)	215 (19 %)	350 (12 %)
Missing	6 (0.3%)	18 (1.6%)	24 (0.8%)
**NICU admission**			
No	1515 (88 %)	980 (86 %)	2495 (87 %)
Yes	136 (8 %)	124 (11 %)	260 (9 %)
Missing	77 (4.5%)	36 (3.2%)	113 (3.9%)
**Gestational Age at Delivery (weeks)**			
Mean (SD)	39 (± 2.1)	39 (± 1.7)	39 (± 2.0)
Missing	0 (0%)	4 (0.4%)	4 (0.1%)
**Infant sex**			
Male	844 (49 %)	563 (49 %)	1407 (49 %)
Female	884 (51 %)	573 (50 %)	1457 (51 %)
Missing	0 (0%)	4 (0.4%)	4 (0.1%)
**Infant birthweight**			
Very low birthweight (<2000g)	70 (4 %)	19 (2 %)	89 (3 %)
Low- moderate birthweight (2000-2500g)	196 (11 %)	39 (3 %)	235 (8 %)
Normal birthweight (>2500g)	1454 (84 %)	1050 (92 %)	2504 (87 %)
Missing	8 (0.5%)	32 (2.8%)	40 (1.4%)
**Age of developmental assessment (months)**			
	12.17 (0.40)	12.39 (0.46)	12.25 (0.44)

346

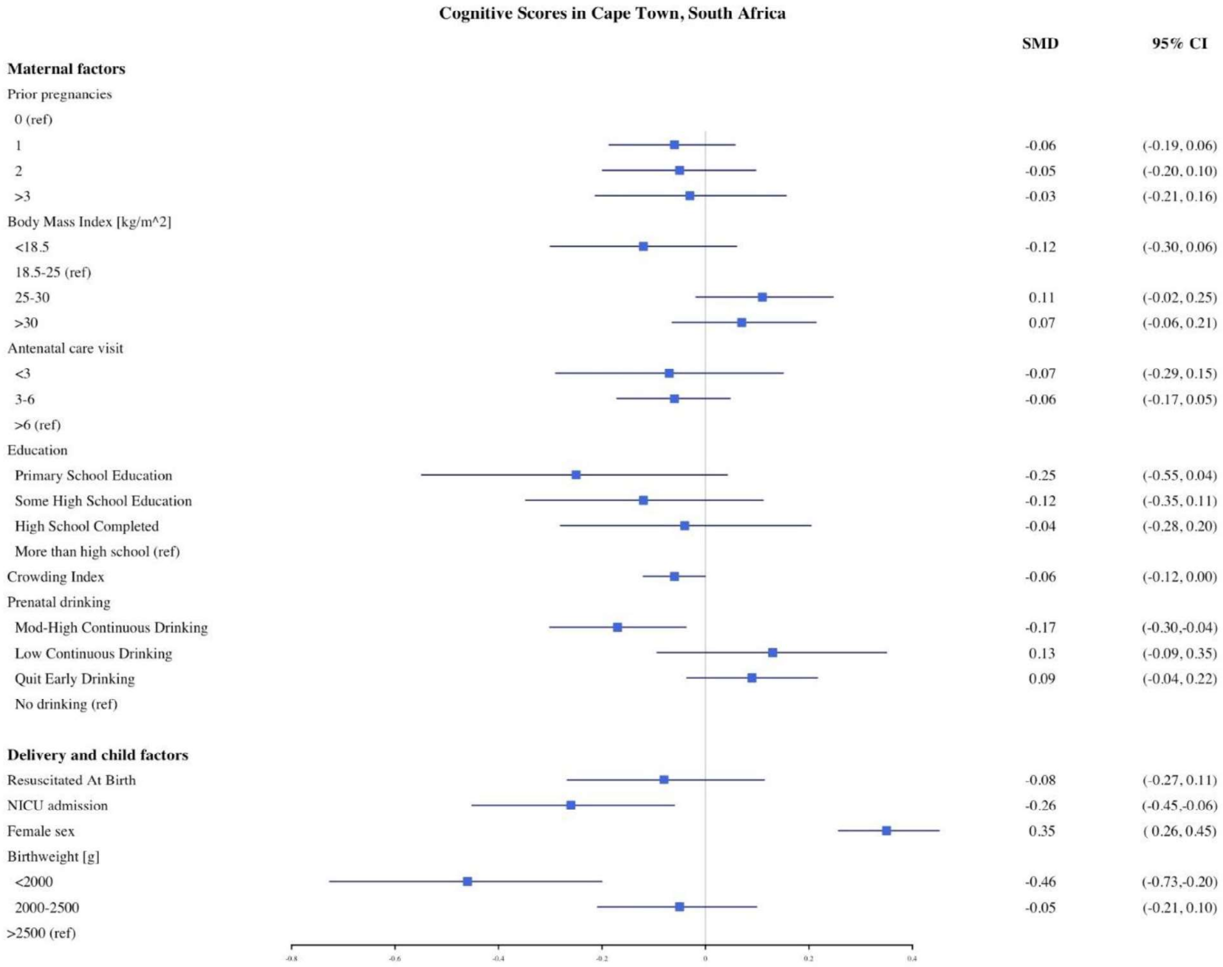

Figure 1: Association of maternal, delivery and child factors with cognitive development in Cape Town, South Africa

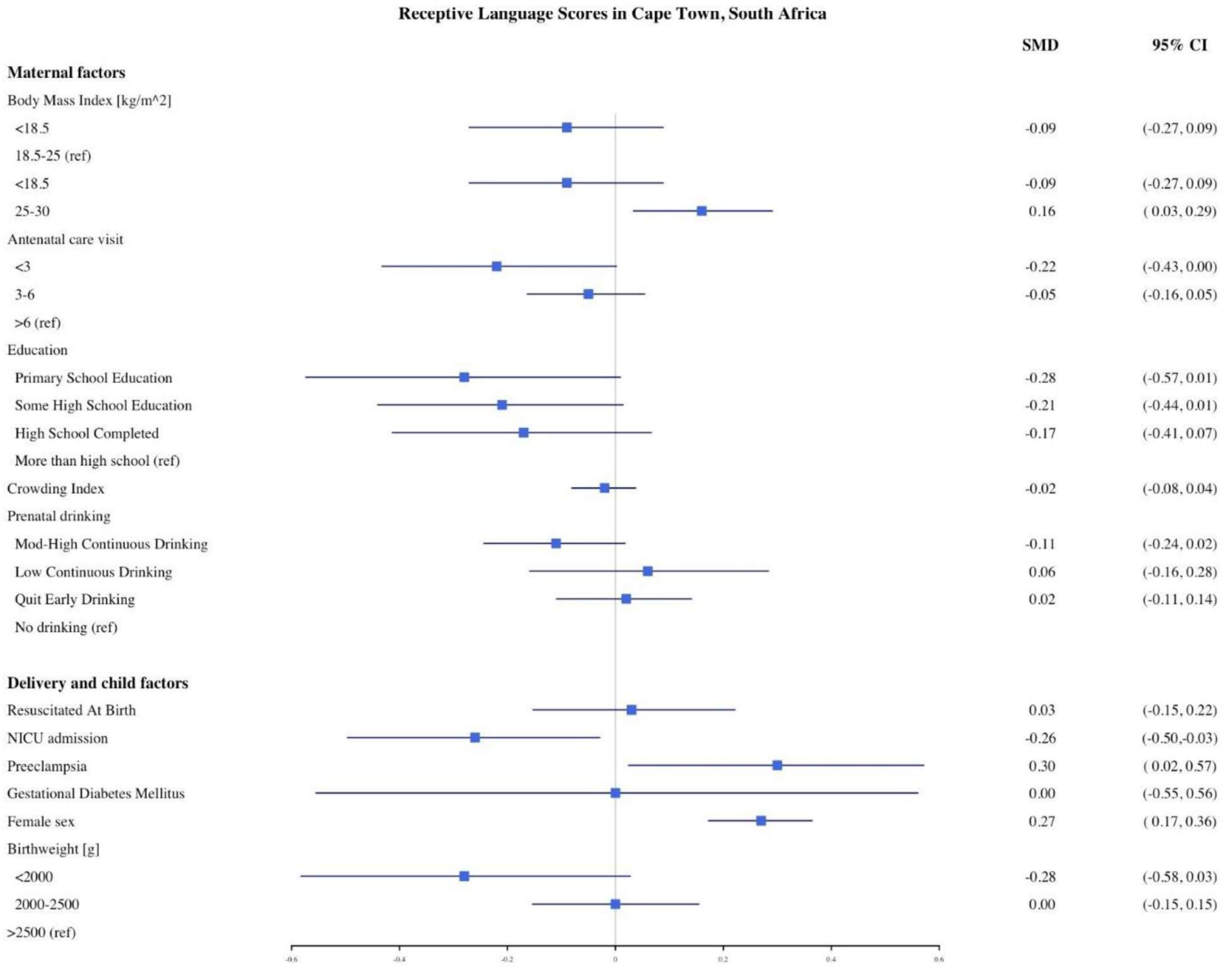

Figure 2: Association of maternal, delivery and child factors with receptive language development in Cape Town, South Africa

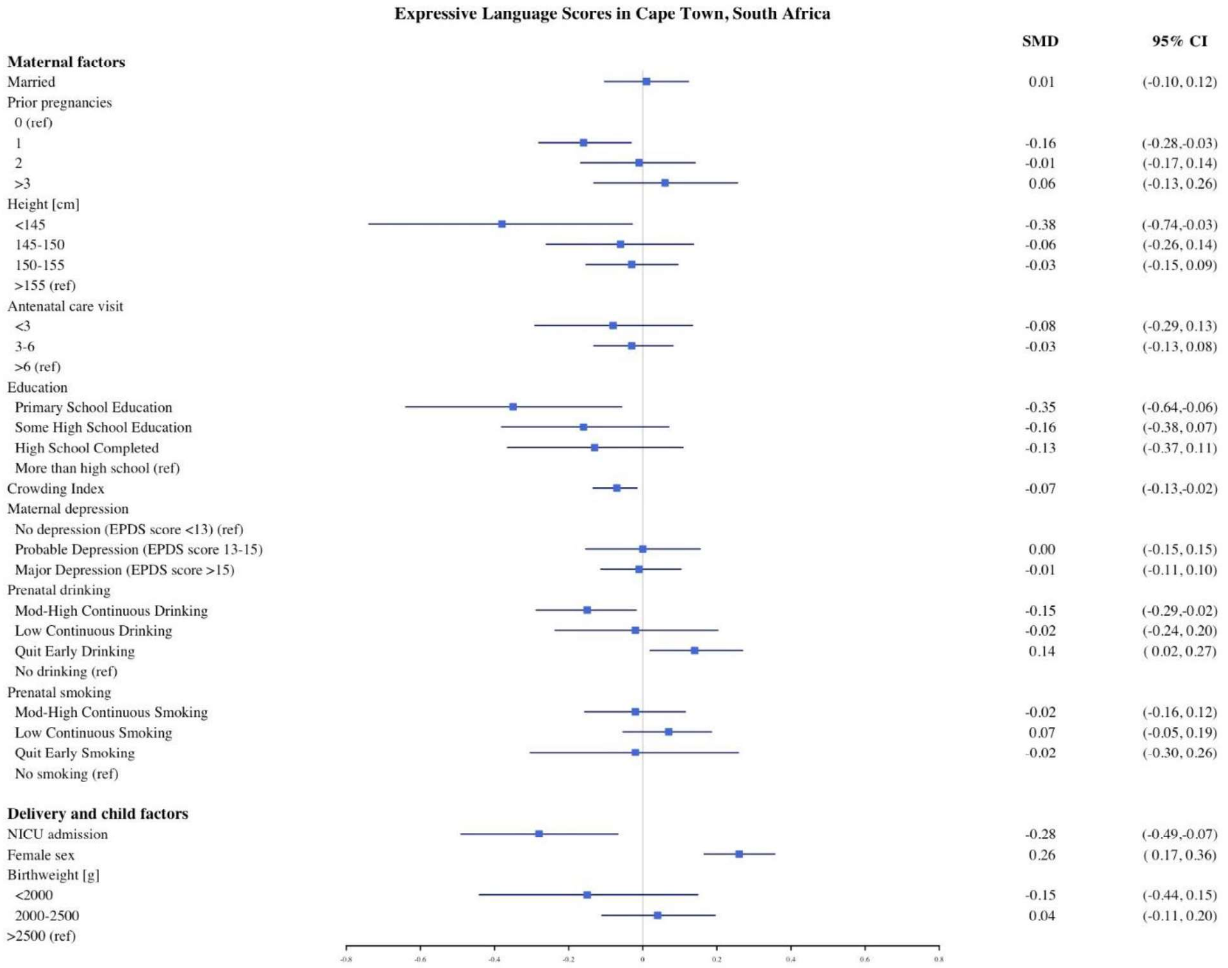

Figure 3: Association of maternal, delivery and child factors with expressive language development in Cape Town, South Africa

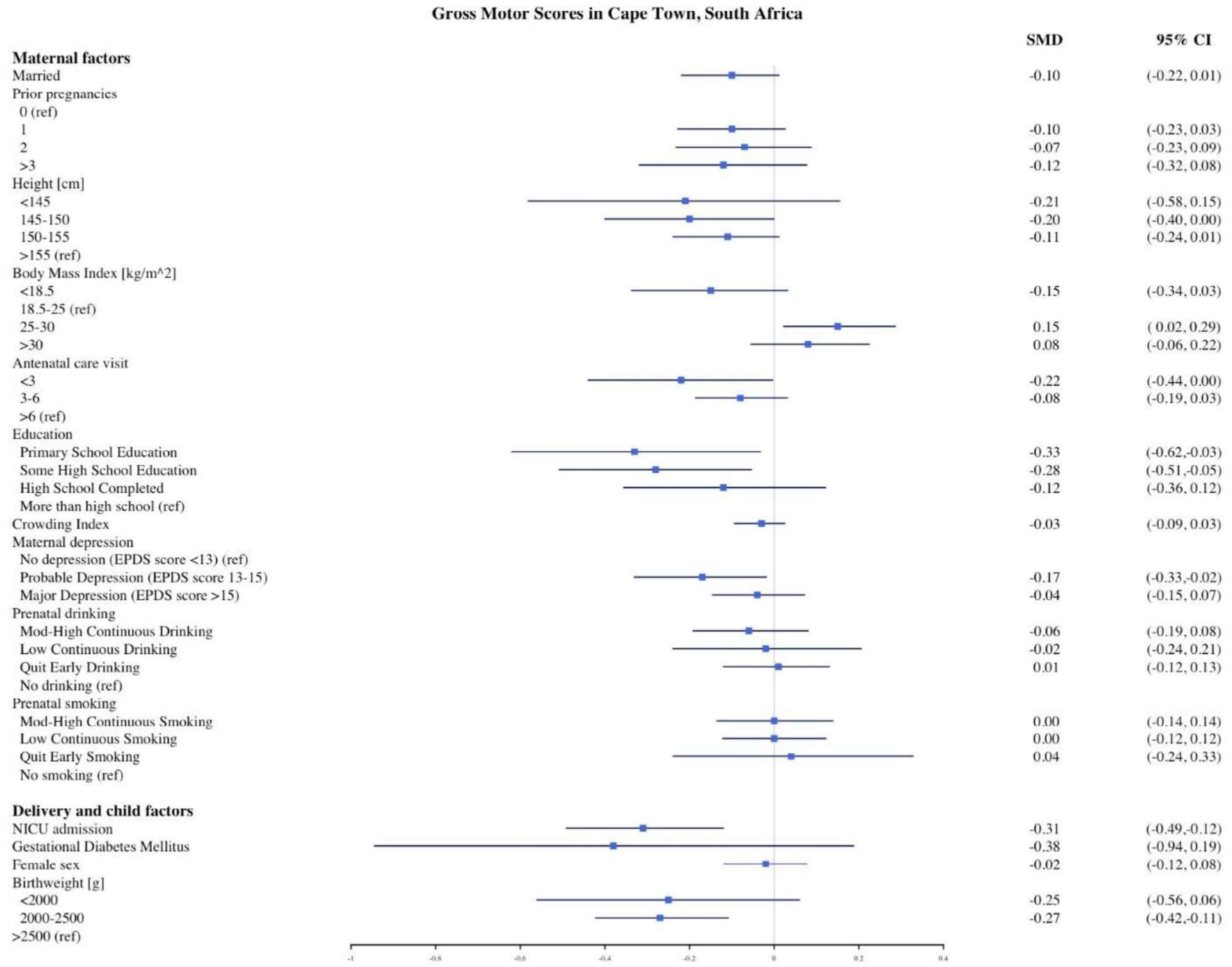

Figure 4: Association of maternal, delivery and child factors with gross motor development in Cape Town, South Africa

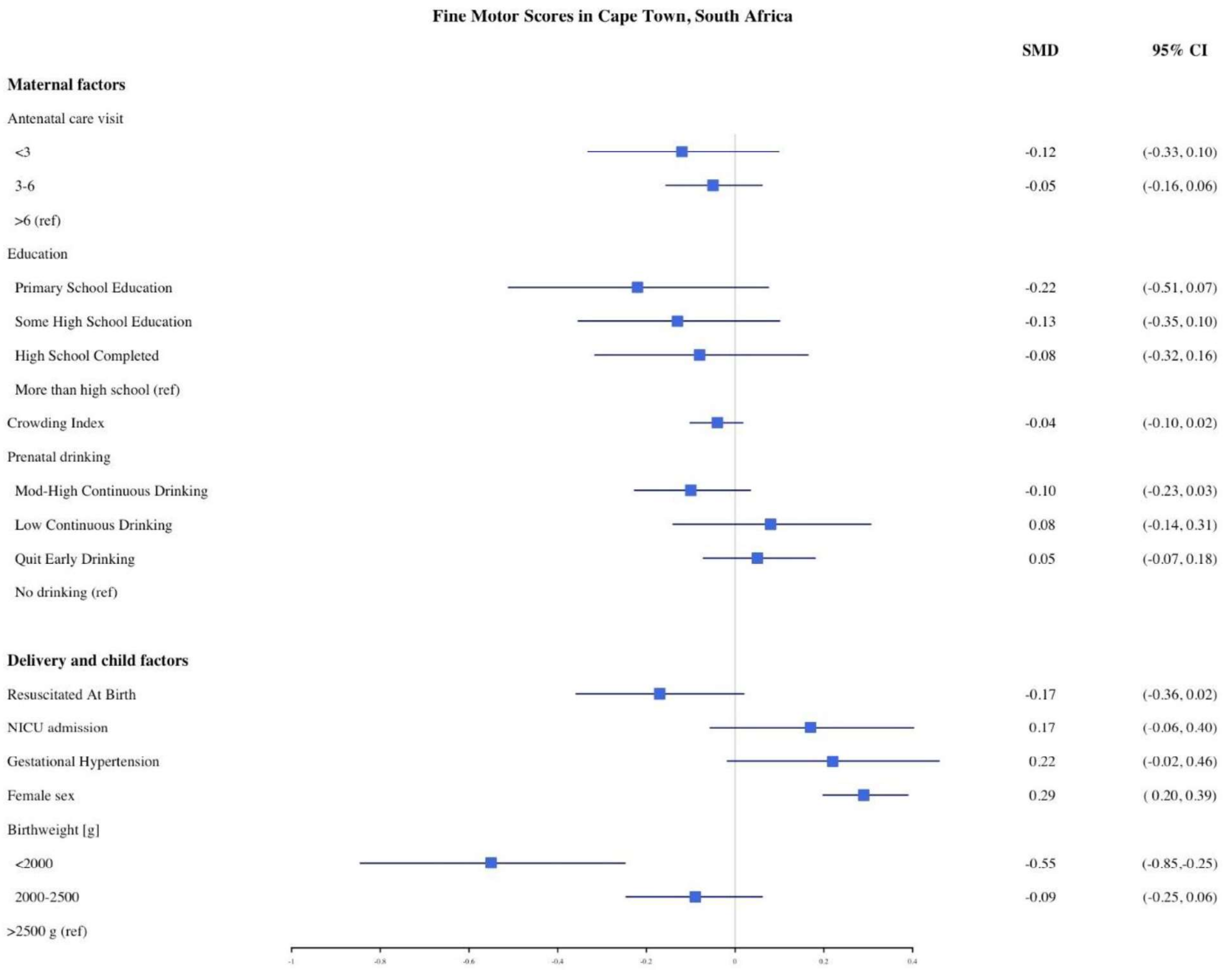

Figure 5: Association of maternal, delivery and child factors with fine motor development in South Africa

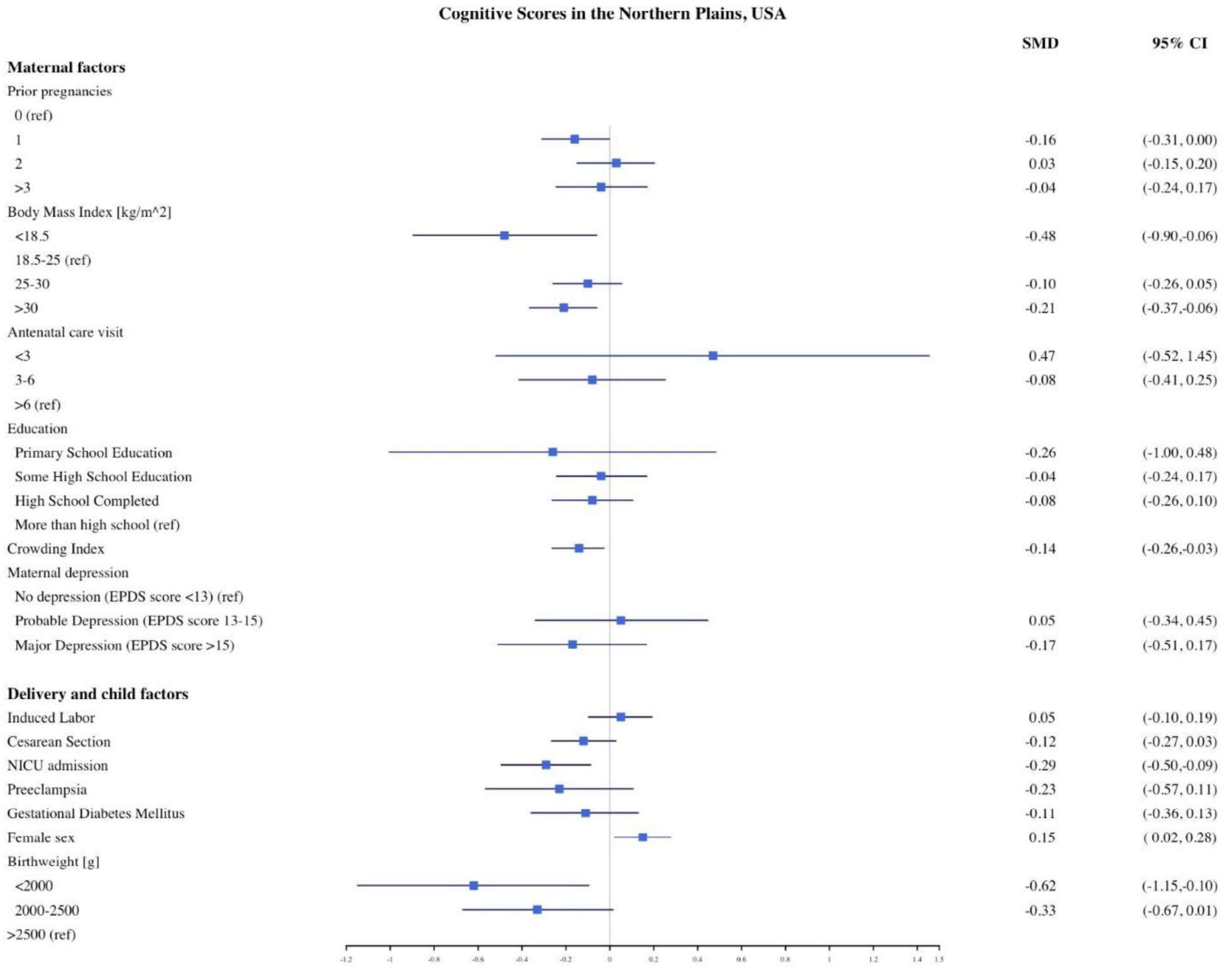

Figure 6: Association of maternal, delivery and child factors with cognitive development in Northern Plains, USA

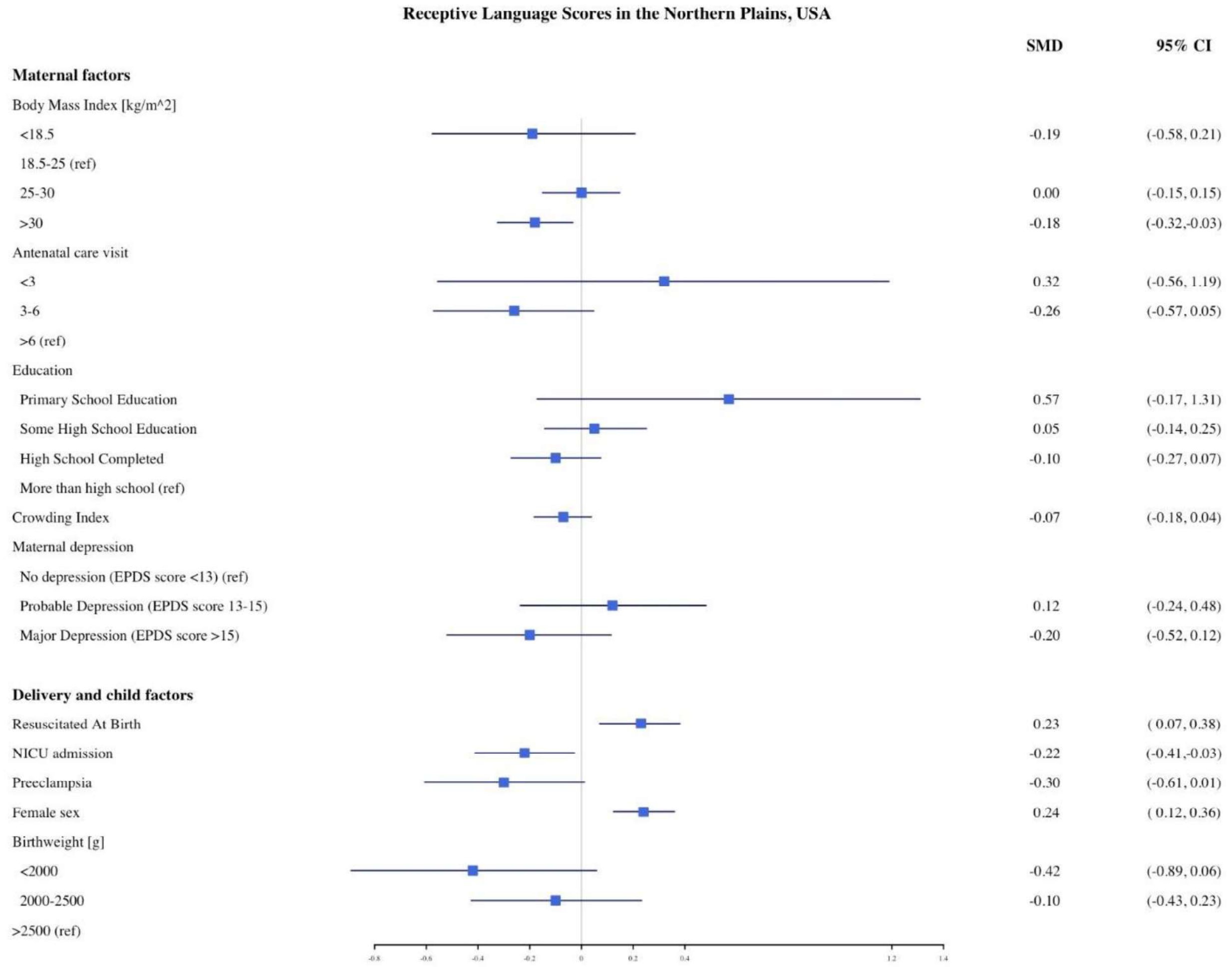

Figure 7: Association of maternal, delivery and child factors with receptive language development in Northern Plains, USA

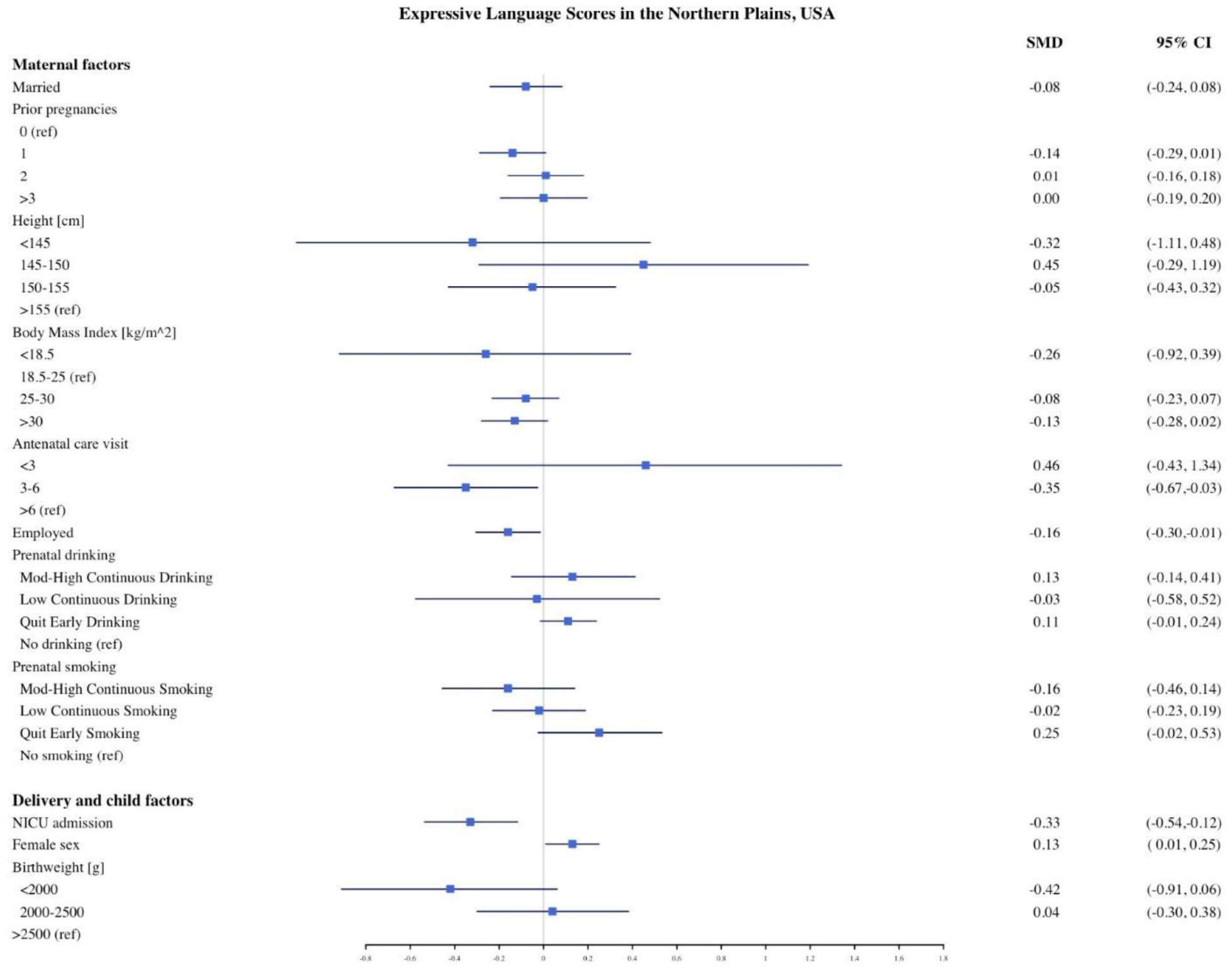

Figure 8: Association of maternal, delivery and child factors with expressive language development in Northern Plains, USA

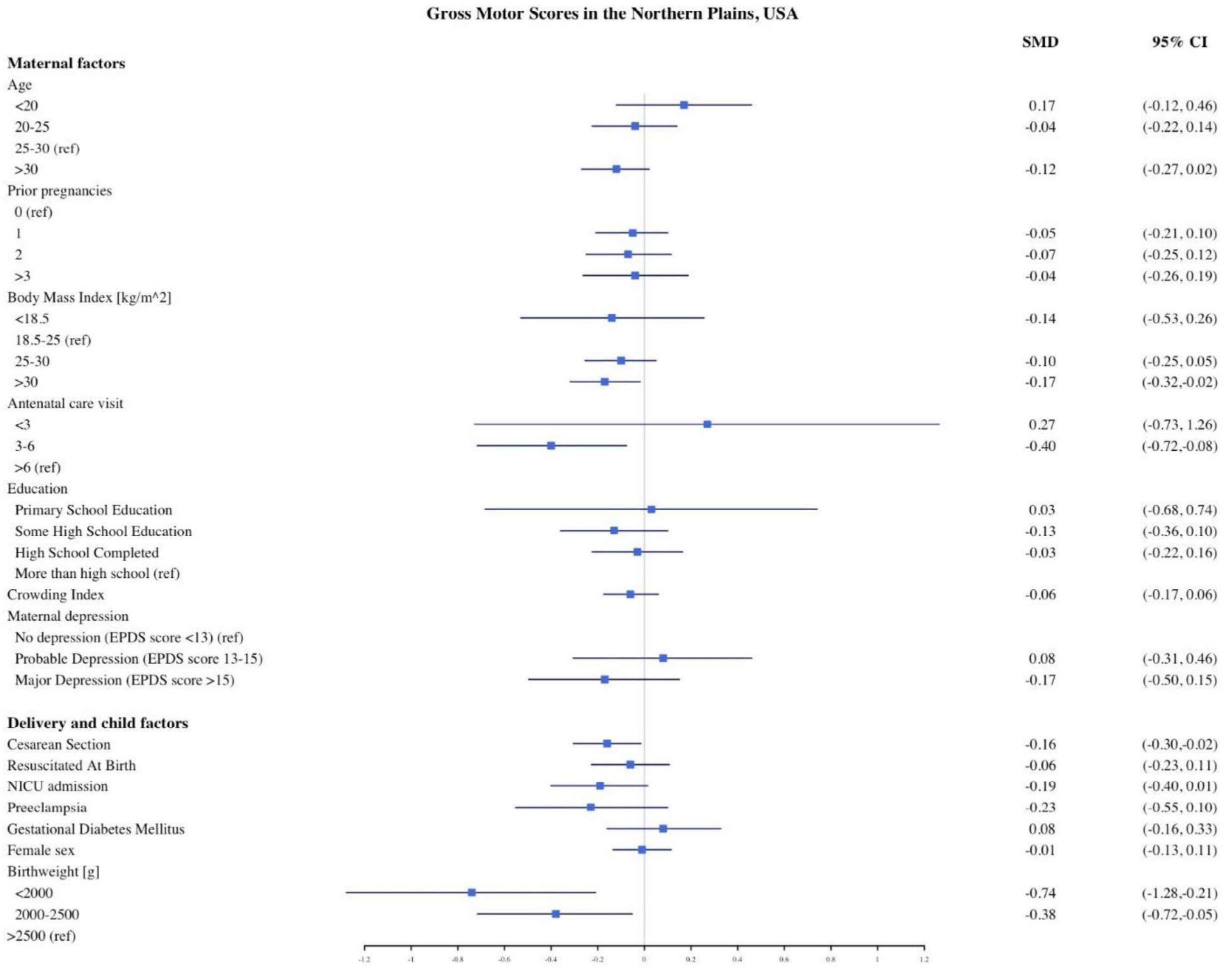

Figure 9: Association of maternal, delivery and child factors with gross motor development in Northern Plains, USA

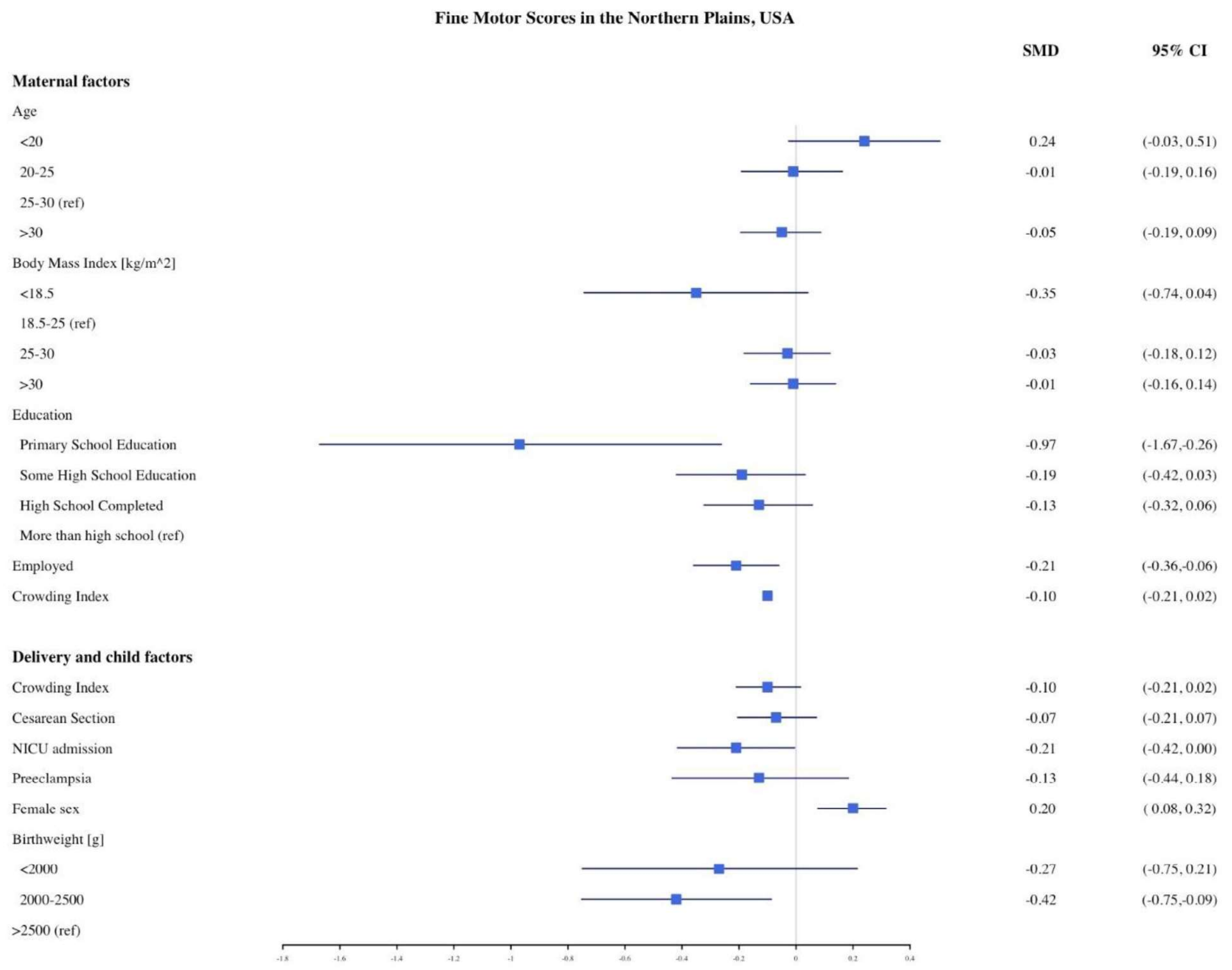

Figure 10: Association of maternal, delivery and child factors with fine motor development in Northern Plains, USA

Supplemental Table S1: Amount smoking and drinking in the cluster groups by trimester

Exposure category	Exposure Information		
	Trimester 1	Trimester 2	Trimester 3
Smoking category	**Average Cigarettes/ week, mean (SD)**		
Non-smokers	0.014 (0.08)	0.003 (0.04)	0.009 (0.06)
Quit early	8.81 (9.92)	0.07 (0.19)	0.11 (0.22)
Low continuous	15.72 (10.28)	15.40 (10.79)	14.33 (10.32)
Moderate to high continuous	48.31 (21.71)	51.71 (25.53)	47.09 (25.29)
Drinking category	**Drinks/ trimester, mean (SD)**		
Non-drinkers	0.04 (0.16)	0.02 (0.10)	0.01 (0.07)
Quit early	8.77 (7.41)	0.12 (0.58)	0.04 (0.26)
Low continuous	2.41 (3.83)	2.27 (2.71)	1.85 (1.25)
Moderate to high continuous	40.89 (60.08)	16.12 (28.27)	7.46 (16.88)

1. McCoy DC, Peet ED, Ezzati M, Danaei G, Black MM, Sudfeld CR, et al. Early Childhood Developmental Status in Low- and Middle-Income Countries: National, Regional, and Global Prevalence Estimates Using Predictive Modeling. PLoS medicine. 2016;13(6):e1002034.
2. Ozawa S, Laing SK, Higgins CR, Yemeke TT, Park CC, Carlson R, et al. Educational and economic returns to cognitive ability in low- and middle-income countries: A systematic review. World development. 2022;149:105668.
3. Sania A, Sudfeld CR, Danaei G, Fink G, McCoy DC, Zhu Z, et al. Early life risk factors of motor, cognitive and language development: a pooled analysis of studies from low/middle-income countries. BMJ open. 2019;9(10):e026449.
4. Walker SP, Wachs TD, Meeks Gardner J, Lozoff B, Wasserman GA, Pollitt E, et al. Child development: risk factors for adverse outcomes in developing countries. The Lancet. 2007;369(9556):145-57.
5. Walker SP, Wachs TD, Grantham-McGregor S, Black MM, Nelson CA, Huffman SL, et al. Inequality in early childhood: risk and protective factors for early child development. Lancet (London, England). 2011;378(9799):1325-38.
6. Fruh V, Rifas-Shiman SL, Amarasiriwardena C, Cardenas A, Bellinger DC, Wise LA, et al. Prenatal lead exposure and childhood executive function and behavioral difficulties in project viva. NeuroToxicology. 2019;75:105-15.
7. McCormack C, Hutchinson D, Burns L, Youssef G, Wilson J, Elliott E, et al. Maternal and partner prenatal alcohol use and infant cognitive development. Drug and alcohol dependence. 2018;185:330-8.
8. Alati R, Davey Smith G, Lewis SJ, Sayal K, Draper ES, Golding J, et al. Effect of prenatal alcohol exposure on childhood academic outcomes: contrasting maternal and paternal associations in the ALSPAC study. PloS one. 2013;8(10):e74844.

9. Hutchinson D, Youssef GJ, McCormack C, Wilson J, Allsop S, Najman J, et al. Prenatal alcohol exposure and infant gross motor development: a prospective cohort study. BMC Pediatrics. 2019;19(1):149.
10. Sanou AS, Diallo AH, Holding P, Nankabirwa V, Engebretsen IMS, Ndeezi G, et al. Maternal alcohol consumption during pregnancy and child's cognitive performance at 6-8 years of age in rural Burkina Faso: an observational study. PeerJ. 2017;5:e3507-e.
11. Sanou AS, Diallo AH, Holding P, Nankabirwa V, Engebretsen IMS, Ndeezi G, et al. Maternal alcohol consumption during pregnancy and child's cognitive performance at 6-8 years of age in rural Burkina Faso: an observational study. PeerJ. 2017;5:e3507.
12. Fan J, Jacobson SW, Taylor PA, Molteno CD, Dodge NC, Stanton ME, et al. White matter deficits mediate effects of prenatal alcohol exposure on cognitive development in childhood. Human brain mapping. 2016;37(8):2943-58.
13. Willford J, Leech S, Day N. Moderate prenatal alcohol exposure and cognitive status of children at age 10. Alcoholism, clinical and experimental research. 2006;30(6):1051-9.
14. Williams Brown C, Carmichael Olson H, Croninger RG. Maternal Alcohol Consumption During Pregnancy and Infant Social, Mental, and Motor Development. 2010;32(2):110-26.
15. Kelly Y, Sacker A, Gray R, Kelly J, Wolke D, Quigley MA. Light drinking in pregnancy, a risk for behavioural problems and cognitive deficits at 3 years of age? International journal of epidemiology. 2009;38(1):129-40.
16. Zuccolo L, Lewis SJ, Davey Smith G, Sayal K, Draper ES, Fraser R, et al. Prenatal alcohol exposure and offspring cognition and school performance. A 'Mendelian randomization' natural experiment. International journal of epidemiology. 2013;42(5):1358-70.
17. May PA, Gossage JP, Marais AS, Hendricks LS, Snell CL, Tabachnick BG, et al. Maternal risk factors for fetal alcohol syndrome and partial fetal alcohol syndrome in South Africa: a third study. Alcoholism, clinical and experimental research. 2008;32(5):738-53.
18. Li C, Zhu N, Zeng L, Dang S, Zhou J, Pei L, et al. Effect of maternal pre-pregnancy underweight and average gestational weight gain on physical growth and intellectual development of early school-aged children. Scientific Reports. 2018;8(1):12014.
19. Hinkle SN, Schieve LA, Stein AD, Swan DW, Ramakrishnan U, Sharma AJ. Associations between maternal prepregnancy body mass index and child neurodevelopment at 2 years of age. International journal of obesity (2005). 2012;36(10):1312-9.
20. Yeung EH, Sundaram R, Ghassabian A, Xie Y, Buck Louis G. Parental Obesity and Early Childhood Development. Pediatrics. 2017;139(2):e20161459.
21. Jo H, Schieve LA, Sharma AJ, Hinkle SN, Li R, Lind JN. Maternal prepregnancy body mass index and child psychosocial development at 6 years of age. Pediatrics. 2015;135(5):e1198-e209.
22. Duffany KO, McVeigh KH, Kershaw TS, Lipkind HS, Ickovics JR. Maternal Obesity: Risks for Developmental Delays in Early Childhood. Maternal and child health journal. 2016;20(2):219-30.
23. Álvarez-Bueno C, Cavero-Redondo I, Lucas-de la Cruz L, Notario-Pacheco B, Martínez-Vizcaíno V. Association between pre-pregnancy overweight and obesity and children's neurocognitive development: a systematic review and meta-analysis of observational studies. International journal of epidemiology. 2017;46(5):1653-66.
24. Sanchez CE, Barry C, Sabhlok A, Russell K, Majors A, Kollins SH, et al. Maternal pre-pregnancy obesity and child neurodevelopmental outcomes: a meta-analysis. Obesity reviews : an official journal of the International Association for the Study of Obesity. 2018;19(4):464-84.
25. Dukes KA, Burd L, Elliott AJ, Fifer WP, Folkerth RD, Hankins GD, et al. The safe passage study: design, methods, recruitment, and follow-up approach. Paediatr Perinat Epidemiol. 2014;28(5):455-65.

26. Dukes KA, Burd L, Elliott AJ, Fifer WP, Folkerth RD, Hankins GDV, et al. The safe passage study: design, methods, recruitment, and follow-up approach. Paediatr Perinat Epidemiol. 2014;28(5):455-65.
27. Bornman J, Romski M, Tonsing K, Sevcik R, White R, Barton-Hulsey A, et al. Adapting and translating the Mullen Scales of Early Learning for the South African context. S Afr J Commun Disord. 2018;65(1):e1-e9.
28. Mitchell CM, Croy C, Spicer P, Frankel K, Emde RN. Trajectories of cognitive development among American Indian young children. Developmental psychology. 2011;47(4):991-9.
29. Dukes K, Tripp T, Petersen J, Robinson F, Odendaal H, Elliott A, et al. A modified Timeline Followback assessment to capture alcohol exposure in pregnant women: Application in the Safe Passage Study. Alcohol. 2017;62:17-27.
30. Pini N, Myers MM, Elliott AJ, Shuffrey LC, Lucchini M, Sania A, et al. Cluster Analysis of Alcohol Consumption during Pregnancy in the Safe Passage Study. Annu Int Conf IEEE Eng Med Biol Soc. 2019;2019:1338-41.
31. Sania A, Myers MM, Pini N, Lucchini M, Nugent JD, Shuffrey LC, et al. Prenatal smoking and drinking are associated with altered newborn autonomic functions. Pediatr Res. 2023;93(1):242-52.
32. Shuffrey LC, Myers MM, Isler JR, Lucchini M, Sania A, Pini N, et al. Association Between Prenatal Exposure to Alcohol and Tobacco and Neonatal Brain Activity: Results From the Safe Passage Study. JAMA Netw Open. 2020;3(5):e204714.
33. Lawrie TA, Hofmeyr GJ, de Jager M, Berk M. Validation of the Edinburgh Postnatal Depression Scale on a cohort of South African women. South African medical journal = Suid-Afrikaanse tydskrif vir geneeskunde. 1998;88(10):1340-4.
34. Blakesley RE, Mazumdar S, Dew MA, Houck PR, Tang G, Reynolds CF, 3rd, et al. Comparisons of methods for multiple hypothesis testing in neuropsychological research. Neuropsychology. 2009;23(2):255-64.
35. Greenland S. Analysis goals, error-cost sensitivity, and analysis hacking: Essential considerations in hypothesis testing and multiple comparisons. 2021;35(1):8-23.
36. Rothman KJ. No adjustments are needed for multiple comparisons. Epidemiology (Cambridge, Mass). 1990;1(1):43-6.
37. Mattson SN, Crocker N, Nguyen TT. Fetal alcohol spectrum disorders: neuropsychological and behavioral features. Neuropsychology review. 2011;21(2):81-101.
38. Noland JS, Singer LT, Arendt RE, Minnes S, Short EJ, Bearer CF. Executive functioning in preschool-age children prenatally exposed to alcohol, cocaine, and marijuana. Alcoholism, clinical and experimental research. 2003;27(4):647-56.
39. Louw JG, van Heerden A, Olivier L, Lambrechts T, Broodryk M, Bunge L, et al. Executive Function After Prenatal Alcohol Exposure in Children in a South African Population: Cross-sectional Study. JMIR formative research. 2021;5(7):e20658.
40. Davies L, Dunn M, Chersich M, Urban M, Chetty C, Olivier L, et al. Developmental delay of infants and young children with and without fetal alcohol spectrum disorder in the Northern Cape Province, South Africa. African journal of psychiatry. 2011;14(4):298-305.
41. Skogerbø Å, Kesmodel US, Denny CH, Kjaersgaard MI, Wimberley T, Landrø NI, et al. The effects of low to moderate alcohol consumption and binge drinking in early pregnancy on behaviour in 5-year-old children: a prospective cohort study on 1628 children. BJOG : an international journal of obstetrics and gynaecology. 2013;120(9):1042-50.
42. Flak AL, Su S, Bertrand J, Denny CH, Kesmodel US, Cogswell ME. The association of mild, moderate, and binge prenatal alcohol exposure and child neuropsychological outcomes: a meta-analysis. Alcoholism, clinical and experimental research. 2014;38(1):214-26.
43. Warriner K, Sibiya MN, Reddy P, Napier C. Nutritional status and dietary diversity of pregnant women in rural KwaZulu-Natal, South Africa. 2019;24(1):1-8.

44. De Bruyn CM. Dietary adequacy, variety and diversity and associated factors (anthropometry and socio-economic status) in pregnant women attending the Bishop Lavis MOU in Cape Town: University of Cape Town; 2015.
45. Idrus NM, Breit KR, Thomas JD. Dietary choline levels modify the effects of prenatal alcohol exposure in rats. Neurotoxicology and teratology. 2017;59:43-52.
46. Zeisel SH. What choline metabolism can tell us about the underlying mechanisms of fetal alcohol spectrum disorders. Molecular neurobiology. 2011;44(2):185-91.
47. Leermakers ET, Moreira EM, Kiefte-de Jong JC, Darweesh SK, Visser T, Voortman T, et al. Effects of choline on health across the life course: a systematic review. Nutrition reviews. 2015;73(8):500-22.
48. Oken E, Thompson JW, Rifas-Shiman SL, Vilchuk K, Bogdanovich N, Hameza M, et al. Analysis of Maternal Prenatal Weight and Offspring Cognition and Behavior: Results From the Promotion of Breastfeeding Intervention Trial (PROBIT) Cohort. JAMA Network Open. 2021;4(8):e2121429-e.
49. Alamri E, Bayomy H, Albishi L. Impact of maternal obesity on the cognitive function and mental health of the offspring: a systematic review. Proceedings of the Nutrition Society. 2022;81(OCE1):E49.
50. St-Germain LE, Castellana B, Baltayeva J, Beristain AG. Maternal Obesity and the Uterine Immune Cell Landscape: The Shaping Role of Inflammation. Int J Mol Sci. 2020;21(11):3776.
51. Onubi OJ, Marais D, Aucott L, Okonofua F, Poobalan AS. Maternal obesity in Africa: a systematic review and meta-analysis. Journal of public health (Oxford, England). 2016;38(3):e218-e31.
52. Lopoo LM, London AS. Household Crowding During Childhood and Long-Term Education Outcomes. Demography. 2016;53(3):699-721.
53. Marsh R, Salika T, Crozier S, Robinson S, Cooper C, Godfrey K, et al. The association between crowding within households and behavioural problems in children: Longitudinal data from the Southampton Women's Survey. 2019;33(3):195-203.
54. Gao Y, Zhang L, Kc A, Wang Y, Zou S, Chen C, et al. Housing environment and early childhood development in sub-Saharan Africa: A cross-sectional analysis. PLoS medicine. 2021;18(4):e1003578.
55. Evans G, Ricciuti H, Hope S, Schoon I. Crowding and Cognitive Development: The Mediating Role of Maternal Responsiveness Among 36-Month-Old Children. Environment and Behavior. 2010;42.
56. Jeong J, Pitchik HO, Yousafzai AK. Stimulation Interventions and Parenting in Low- and Middle-Income Countries: A Meta-analysis. Pediatrics. 2018;141(4).
