# Supplementary material for: Maternal obesity and prenatal alcohol exposure are associated with child development: Results from the Safe Passage Study
Source: PLoS One. 2026 Apr 6;21(4):e0345406. doi: 10.1371/journal.pone.0345406 (PMC13052907; doi:10.1371/journal.pone.0345406)
Supplement: Attachment — Submitted filename: response to reviewers.docx [file pone.0345406.s006.docx]

We are thankful to the editor and to the reviewers for the thoughtful comments of our manuscript. We have provided both the highlighted version and a clean version of the revised manuscript. As requested, below, we have provided a response to each editor and reviewer comment (*indicated in italics*) and identified the page and paragraph numbers where the changes (**indicated in bold**) have been made to the clean revised manuscript. Additionally, minor edits were made to the manuscript for consistency, clarification and simplification and are noted in the highlighted version of the revised manuscript.

**Editor:**

*1.Please ensure that your manuscript meets PLOS ONE's style requirements, including those for file naming. The PLOS ONE style templates can be found at*

[*https://journals.plos.org/plosone/s/file?id=wjVg/PLOSOne_formatting_sample_main_body.pdf*](https://journals.plos.org/plosone/s/file?id=wjVg/PLOSOne_formatting_sample_main_body.pdf) *and*

[*https://journals.plos.org/plosone/s/file?id=ba62/PLOSOne_formatting_sample_title_authors_affiliations.pdf*](https://nam02.safelinks.protection.outlook.com/?url=https%3A%2F%2Ftrack.editorialmanager.com%2FCL0%2Fhttps%3A%252F%252Fjournals.plos.org%252Fplosone%252Fs%252Ffile%253Fid%3Dba62%252FPLOSOne_formatting_sample_title_authors_affiliations.pdf%2F1%2F010f01990dc15a9c-32bff0cb-66b6-4548-b23d-170125d983ce-000000%2FgIaNYmCdJNZbH-_kBX7nMI-nX4vkvIEz1hMdn6Y-lCg%3D227&data=05%7C02%7Cas4823%40cumc.columbia.edu%7C5bd290a3c2134352ab0808ddea9f4951%7Cb0002a9b0017404d97dc3d3bab09be81%7C0%7C0%7C638924692447152195%7CUnknown%7CTWFpbGZsb3d8eyJFbXB0eU1hcGkiOnRydWUsIlYiOiIwLjAuMDAwMCIsIlAiOiJXaW4zMiIsIkFOIjoiTWFpbCIsIldUIjoyfQ%3D%3D%7C0%7C%7C%7C&sdata=P6e6A8anp47rmYSKhwccdLvaJxotwlz1qctnUzY9mg0%3D&reserved=0)

**Response:** We have reviewed the guidelines and formatted the manuscript accordingly.

*2.  Thank you for stating the following financial disclosure:*

*“This research was supported by grants UH3OD023279, U01HD055154, U01HD045935, U01HD055155, and U01AA016501, issued by the Office of the Director of the National Institutes of Health, National Institute on Alcohol Abuse and Alcoholism, Eunice Kennedy Shriver National Institute of Child Health and Human Development, and the National Institute on Deafness and Other Communication Disorders. Ayesha Sania was supported by a career development award from the Fogarty International Center at the NIH (1K01TW012425-01A1). The opinions expressed in this paper are those of the authors and do not necessarily represent the official views of the National Institutes of Health, the Eunice Kennedy Shriver National Institute of Child Health and Development, or Fogarty International Center, the National Institute on Alcohol Abuse and Alcoholism, or the National Institute on Deafness and Other Communication Disorders.”*

*Please state what role the funders took in the study.  If the funders had no role, please state: "The funders had no role in study design, data collection and analysis, decision to publish, or preparation of the manuscript." If this statement is not correct you must amend it as needed.*

*Please include this amended Role of Funder statement in your cover letter; we will change the online submission form on your behalf.*

Response: We have added this statement to the cover letter.

*3. We note that you have indicated that there are restrictions to data sharing for this study. PLOS only allows data to be available upon request if there are legal or ethical restrictions on sharing data publicly. For more information on unacceptable data access restrictions, please see* [*http://journals.plos.org/plosone/s/data-availability#loc-unacceptable-data-access-restrictions*](http://journals.plos.org/plosone/s/data-availability#loc-unacceptable-data-access-restrictions)*. Before we proceed with your manuscript, please address the following prompts:*

*a) If there are ethical or legal restrictions on sharing a de-identified data set, please explain them in detail (e.g., data contain potentially identifying or sensitive patient information, data are owned by a third-party organization, etc.) and who has imposed them (e.g., a Research Ethics Committee or Institutional Review Board, etc.). Please also provide contact information for a data access committee, ethics committee, or other institutional body to which data requests may be sent.*

*b) If there are no restrictions, please upload the minimal anonymized data set necessary to replicate your study findings to a stable, public repository and provide us with the relevant URLs, DOIs, or accession numbers. For a list of recommended repositories, please see*

[*https://journals.plos.org/plosone/s/recommended-repositories*](https://journals.plos.org/plosone/s/recommended-repositories)*. You also have the option of uploading the data as Supporting Information files, but we would recommend depositing data directly to a data repository if possible. We will update your Data Availability statement on your behalf to reflect the information you provide.*

**Response: We have updated the data availability statement in the online submission system and in the cover letter. We now provide a location where data can be accessed.**

**Data Availability:** De-identified data from the Safe Passage Study is available through NICHD’s Data and Specimen Hub (DASH). All cases of demographic and exposure data are available on DASH. Elliott, Amy (2025). A Prospective Study on the Role of Prenatal Alcohol Exposure in SIDS and Stillbirth (Version 1). NICHD Data and Specimen Hub. [https://doi.org/10.57982/sv8c-4y07](https://nam02.safelinks.protection.outlook.com/?url=https%3A%2F%2Fdoi.org%2F10.57982%2Fsv8c-4y07&data=05%7C02%7Cas4823%40cumc.columbia.edu%7C932e87bf440b44a2a69608de1c87144e%7Cb0002a9b0017404d97dc3d3bab09be81%7C0%7C0%7C638979564001617858%7CUnknown%7CTWFpbGZsb3d8eyJFbXB0eU1hcGkiOnRydWUsIlYiOiIwLjAuMDAwMCIsIlAiOiJXaW4zMiIsIkFOIjoiTWFpbCIsIldUIjoyfQ%3D%3D%7C0%7C%7C%7C&sdata=gRj44Zcezktvh1xz6g7nzPA%2F7NvH%2FFJM2wil7JLJGBA%3D&reserved=0). The tribal data used in this study is restricted access per the requirements of participating tribal nations and the Indian Health Service IRB. Avera Health maintains the data on a secure server, and people can contact Dr. Christine Hockett ([Christine.hockett@avera.org](mailto:Christine.hockett@avera.org)) to learn the process for gaining tribal approval and the necessary regulatory approvals to gain access.

*4. Please include your full ethics statement in the ‘Methods’ section of your manuscript file. In your statement, please include the full name of the IRB or ethics committee who approved or waived your study, as well as whether or not you obtained informed written or verbal consent. If consent was waived for your study, please include this information in your statement as well.*

Response: We now include full names of the IRB in the methods section **(Page 8, Paragraph 2)**

*5. If the reviewer comments include a recommendation to cite specific previously published works, please review and evaluate these publications to determine whether they are relevant and should be cited. There is no requirement to cite these works unless the editor has indicated otherwise.* 

Response: Thank you for this guidance. We have reviewed the articles proposed by reviewers and included the relevant ones.

*6. Please review your reference list to ensure that it is complete and correct. If you have cited papers that have been retracted, please include the rationale for doing so in the manuscript text, or remove these references and replace them with relevant current references. Any changes to the reference list should be mentioned in the rebuttal letter that accompanies your revised manuscript. If you need to cite a retracted article, indicate the article’s retracted status in the References list and also include a citation and full reference for the retraction notice.*

Response: We have reviewed the reference list and updated to PLOS citation style

***Reviewer #1:***

*The study aimed to evaluate cognitive, motor, and language development of 1-year old children by using the Mullen Scales of Early Learning in a prospective cohort study of mother-infant dyads from Northern Plains (NP), USA and Cape Town (CT), South Africa. The relative contributions of prenatal alcohol use, maternal socioeconomic, and nutritional status were investigated within each study site.

The study presents the results of original research. Strengths include the prospective design, the large study sample, inclusion of both high- and low-income settings and measurements by trained assessors using standardized methods. Limitations include assessors not blinded (?) to predictor variables and the short duration of follow-up. I think these should be addressed in the Discussion section.

I have some detailed comments that follow below:

ABSTRACT
1. In the abstract, data from Northern Plains (NP) are presented first, however results starts with Cape Town (CT). I would consistently refer to CT first, then NP – throughout the abstract and manuscript*

Response: Thank you for pointing out this. We have revised the abstract and throughout the manuscript to present results from Cape Town first.

*2. In the abstract, only prenatal alcohol use, maternal socioeconomic and nutritional status are mentioned in the first paragraph, while prenatal and delivery factors are mentioned in the second paragraph, perhaps mention the three main statistical models, i.e. maternal (or prenatal) factors, pregnancy complications and child factors?*

Response: We have revised the abstract. We now mention the three models in the methods part of the abstract (**Page 2).**

*INTRODUCTION
1. I was surprised about the potential positive effects of alcohol on cognition in high income settings, and I think the phrasing of the sentence could be misleading, even though the authors explain that it may be an effect of socioeconomic status. There are other references stating the harmful effects of alcohol also in high-income settings, for instance:
a. Hoyme HE, Kalberg WO, Elliott AJ, Blankenship J, Buckley D, Marais AS et al. Updated clinical guidelines for diagnosing fetal Alcohol Spectrum disorders. Pediatrics. 2016;138(2).
b. Lange S, Probst C, Gmel G, Rehm J, Burd L, Popova S. Global prevalence of fetal alcohol spectrum disorder among children and youth: a systematic review and Meta-analysis. JAMA Pediatr. 2017;171(10):948–56.
c. Jacobson JL, Akkaya-Hocagil T, Ryan LM, Dodge NC, Richardson GA, Olson HC, et al. Effects of prenatal alcohol exposure on cognitive and behavioral development: findings from a hierarchical meta-analysis of data from six prospective longitudinal U.S. cohorts. Alcohol Clin Exp Res. 2021;45(10):2040–58*

Response: We have added a statement about harmful effects of drinking in high income settings and cited the Jacobson et al and Lange et al papers. **(Page 3, Paragraph 2)**

*METHODS
2. Page 7, line 173-175: Are maternal socioeconomic indicators included in both Model 1 and 2?*

Response: We clarified that all models include maternal socioeconomic condition. **(Page 8, Paragraph 1)**

*3. Were the trained assessors performing the Mullen blinded to the predictive factors? That would be a strength that could be emphasized. In case they were not blinded this potential bias should be discussed in Discussion.*

Response: We now mention in the method section that the assessors were blinded of the exposure status. **(Page 6, Paragraph 1)**

*RESULTS
4. I would be consistent throughout the manuscript starting with the CT results*

**Response:** Thank you for this helpful suggestion. We have revised the Results section to consistently present findings from the Cape Town site first throughout the manuscript. In the Discussion, results are organized thematically to support interpretation and synthesis across sites rather than strictly by site order.

*5. Page 8, line 198-199: Perhaps use the abbreviations CT and NP once they are introduced?*

Response: Thank you. We have made these changes.

*6. I was also curious to know which factors contributed the most to developmental outcomes, the mother or the child factors – perhaps this could be discussed in the Discussion*

**Response:** We appreciate this thoughtful suggestion. Our analyses were designed to estimate associations between individual prenatal and contextual risk factors and developmental outcomes, rather than to compare their relative population-level contributions. Direct comparison of effect sizes across maternal and child factors would require consideration of exposure prevalence and potentially estimation of population attributable risk, which was beyond the scope of the present study.

*DISCUSSION
7. Also here I would be consistent starting with CT*

**Response:** We have revised the Discussion to present Cape Town findings first. In instances where cross-site comparisons are discussed thematically, we structured the text to optimize interpretability and conceptual flow rather than adhere strictly to site order.

*8. I would address limitations such as assessors not being blinded to predictors (if that was the case) and the limited follow-up period of 1 year – as children are in continuous development, and 1 year is too early to determine any* long-term effects

**Response:** Thank you for this important comment. We have revised the Discussion to explicitly acknowledge the limited duration of follow-up and clarify that developmental outcomes at 1 year represent early indicators rather than long-term effects. **(Page 17, Paragraph 2).**

*FIGURES AND TABLES
9. Is there a typo in Table 1: Northern Plaines? (should be Northern Plains*)

Response: We have corrected this typo. Thank you.

**Reviewer #2:**

*SUMMARY:
This is a secondary analysis of a large cohort study of mother-infant dyads in two areas with high rates of maternal alcohol use. It assesses infant developmental outcomes at 1 year of age in relation to a variety of demographic, prenatal and perinatal exposures using multiple logistic regression analysis controlling for various confounding variables in 3 categories. A number of known demographic and perinatal risk factors for poor developmental outcomes were confirmed to be present in both cohorts. There were many differences in exposures between the Cape Town and Northern Plains sites, though the sites were not compared statistically, with the analysis being conducted internally for each site. For example prenatal alcohol and smoking exposures were much more common in the Cape Town site, and obesity more common in the Northern Plains site. High and ongoing prenatal alcohol exposure with associated with poor developmental outcome in the CT site but not the NP site, whereas the reverse was true for maternal obesity. Prenatal smoking exposure was not associated with poor developmental outcome. The authors concluded that risk factors could potentially be used to identify infants for early developmental interventions, but that these should context specific given the differences found between sites.

MAJOR COMMENTS:
1. The study is a well designed large cohort study. The cohort, studies methods and primary outcomes have been described in other articles. The findings presented in this paper do not appear to have been published elsewhere and are interesting in themselves. It is stated (line 106) that a subset of women enrolled before 24 weeks were randomly selected for further procedures. Please indicate briefly the process of selection.*

Response: Thank you for this comment. We have clarified the random selection process in the Methods section, including the timing and eligibility criteria for inclusion in the embedded study. Specifically, approximately one in three eligible and consenting women enrolled before 24 weeks’ gestation was randomly selected at the recruitment interview to participate in additional postnatal assessments (Page 5, Paragraph 1). We also added a study flow chart to illustrate the derivation of the final analytic sample (**Supplemental Figure 1).**

*2. At the beginning of the introduction, it is mentioned that early cognitive... development is a key determinant of future educational and income achievement (line 54-55). It is not discussed how best to measure this and it should be discussed to what extent developmental outcomes at 1 year are sufficient for this purpose.*

**Response:** Thank you for this important point. We have clarified in the Discussion that developmental assessment at 1 year captures early neurodevelopmental function but may not fully reflect later-emerging cognitive, behavioral, and academic outcomes and longer-term follow-up would allow examination of a broader range of developmental domains **(Page 17, Paragraph 2).**

*3. The introduction mentions that PAE is more risky in low income settings (line 69) than high income settings and various potential protective factors in high income settings are mentioned. The role of additional risk factors in low income settings should also be mentioned e.g. binge drinking is known to be a common pattern in South Africa.*

**Response:** Thank you for this helpful suggestion. We have revised the Introduction to mention the contextual differences in exposure patterns (Page 3, Paragraph 2). We expanded the Discussion and refer readers to Supplemental Table 2, which describes differences in binge drinking patterns across sites **(Page 15, paragraph 2).**
 *4. In the methods section on Study Populations (line 96-109), little background is given regarding the nature of the two study sites. Some further context would help readers to interpret the article e.g. are the residential areas urban vs rural, how do SES generally compare to average in each country?*

**Response:** Thank you for this helpful suggestion. We have revised the Methods section to provide additional context regarding the study sites, including the specific residential areas in the Western Cape (Bishop Lavis and Belhar in Cape Town) and the geographic composition of the Northern Plains sites, rural and reservation communities in North Dakota and South Dakota. **(Page 5, Paragraph 1).**

*5. 3750 infants were selected for further procedures (line 106), and 2868 (line 193) were included in this study (i.e. 24% dropouts). Please specify the reasons for dropouts per study site.*

**Response:** Thank you for this important point. We added a flow chart summarizing participant selection and exclusions by study site. Participants were recruited during pregnancy and causes of reductions in sample included fetal loss, stillbirth, infant deaths, loss to follow up and missing developmental assessment visit. **(Supplemental Figure 1).**
*6. The manner in which alcohol and smoking exposures were stratified was described in a previous paper by Dukes et al (2017) and is statistically sophisticated. It allows for assessment of longitudinal drinking and smoking practices through pregnancy, but makes it less obvious to the reader how risky is the drinking at any particular timepoint. Details of drinking and smoking are describe for the cohort as a whole in supplementary table 1. It would be helpful to include rates of binge drinking per trimester, since binge drinking is a specific risk for adverse outcomes of PAE. This could be included in Supplementary Table 1 and/or text, and does not require additional statistical analysis. Given that the analyses are done for the two sites independently, Supplementary Table 1 should give drinking and smoking for the two sites separately. Also, given that substance exposures are a particular point of interest for the Safe Passage study it should be stated why other drug use is not included in the analysis.*

**Response:** Thank you for this thoughtful comment. We have added a supplementary table reporting rates of binge drinking per trimester by exposure cluster and stratified by study site (Supplemental Table 2). Drinking and smoking clusters were derived from analysis combining sites, consistent with the previously published approach. For this reason, cluster definitions are presented overall. However, the proportion of exposure clusters by study site is provided in Table 1 to allow site-specific interpretation. Other drug use was reported by approximately 10% of participants when combining multiple drug categories. However, this variable did not meet the pre-specified inclusion threshold (p < 0.20) in minimally adjusted analyses and therefore, other drug use was not included in the multivariable models. We have clarified this in the Methods section **(Page 7, Paragraph 3).**

*7. On lines 145-6, it is stated that the standard number of drinks of alcohol consumed per day of pregnancy was estimated. This is expressed in Supplementary table 1 as the drinks/trimester. Therefore perhaps better to state here also drinks/trimester rather than drinks/day.*

**Response:** Thank you for this suggestion. Although alcohol consumption was recorded at the daily level using the timeline follow-back method, daily estimates were aggregated to trimester-level averages for clustering and reporting. We have clarified this in the Methods section **(Page 7, Paragraph 1).**

*8. Figures 1-10 outline neurodevelopmental outcomes at each site by risk factor. These are presented as SMDs with 95% CIs. P-values are not included, and should be included. The risk factors included vary considerably between figures - please indicate in the text why this is the case and try to standardize as much as possible which variables are included and try to at least include smoking in all figures given that it is a stated point of interest.*

**Response:** Thank you for this helpful comment. We have revised all figures to include p-values alongside standardized mean differences and 95% confidence intervals. The set of risk factors included in the multivariable models varied across outcomes because we applied a pre-specified variable selection procedure. Specifically, we first fit minimally adjusted models (adjusting for child age and sex), and only variables meeting the screening threshold of p < 0.20 were included in the multivariable model for that outcome and site. Smoking did not meet this threshold in the univariate analyses for any outcome and was therefore not included in the final multivariable models. We have clarified this in the Methods section **(Page 7, Paragraph 3).**
*9. It is stated in the text (line 217) that in CT probable depression was associated with lower gross motor scores than no depression. It think this should be tempered with a statement that there was a big group with major depression and this was not associated with lower gross motor score (so there is not apparent "dose-response" effect).*

**Response:** Thank you for this important observation. We have revised the Results section to clarify that although probable depression (EPDS 13–15) was associated with lower gross motor scores, the group with EPDS scores >15 (major depression) did not differ significantly from the reference group. **(Page 12, Paragraph 1).**

*10. In the discussion, it is mentioned that multiple factors classically associated with child development showed similar effects across sites (lines 269-272). Child sex should be included on the list (as an aside, the effect of child sex seems particular large in the CT site, though this is probably hard to interpret). You end up proposing variables to identify infants for early intervention - should child sex be one of these?*

**Response:** We thank the reviewer for this important question. While we observed consistent sex differences in cognitive, language and fine motor outcomes across both study sites, we do not propose sex as a screening marker. Sex differences in early development are well documented and likely reflect biological differences in maturation timing rather than modifiable risk.

*11. In relation to the fact that PAE affected developmental outcome in CT but not NP you indicate (line 287) that residual confounding is unlikely due to a uniform set of confounding variables being assessed. Is it not possible that unmeasured confounder/s could have affected results? (e.g. drug use, for argument sake). In addition, there is evidence that the blood alcohol concentration is an important determinant of risk related to PAE - BAC is determined not just by the level of alcohol consumption but also by volume of distribution/body size (women in NP were bigger - likely reducing effect of PAE), and drinking behavior that was not described (e.g. binges). It would therefore be appropriate to add some further nuance to the discussion of dietary deficiencies (line 289-295).*

**Response:** We thank the reviewer for this thoughtful and important comment. We have revised the Discussion to provide greater nuance regarding potential explanations for the differential findings across sites. We now note that binge drinking patterns were more prevalent in the Cape Town site **(Supplemental Table 2),** which may have resulted in higher peak blood alcohol concentrations. **(Page 15, Paragraph 2)**

*12. Maternal obesity was associated with developmental outcome in NP but not CT. It is suggest that this may be due to lower levels of obesity in CT or due to measurement error in CT due to maternal weight being measured on average at 20 weeks vs 16 weeks (average gestations at enrollment) (lines 304-308). I find this unconvincing - a considerable number of individuals in CT measured as obese, and the 4 week difference does not feel obviously sufficient to mask an effect. It seems equally plausible that other explanations are possible (though speculative) e.g. in an environment where dietary deficiencies are more common obesity may be associated with less deficiency of specific nutrients.*

**Response:** We thank the reviewer for this thoughtful comment. We agree that differences in obesity prevalence or timing of BMI measurement alone may not fully explain the null association observed in the Cape Town site., We now note that in low-resource settings, higher maternal BMI may not solely reflect excess caloric intake, as overweight and obesity frequently coexist with micronutrient deficiencies among women of reproductive age. We cite supporting literature on the double burden of malnutrition and discuss how this nutritional context may influence the association between maternal obesity and child developmental outcomes **(Page 16, Paragraph 2).**

*13. The fact that this is a secondary analysis means there should be some caution about the results. This is especially true given the large number of statistical comparisons and the lack of statistical adjustment for this. For example it is not obvious why pre-eclampsia would be associated with a better receptive language score in CT (figure 2), or why resuscitation at birth would be associated with better outcome in NP (figure 7) and this result could perhaps be the result of multiple comparisons? The role of multiple comparisons should be noted in the limitations, which should be stated in more detail. The use of developmental outcomes at 1 year is an imperfect proxy for long term development (outcome at 3 or 5 years of age would be better) - this is also a limitation to be mentioned.*

Response: We thank the reviewer for these thoughtful comments. We did not apply formal correction methods because the developmental outcomes and covariates are correlated(1-3). We have strengthened the limitations section to explicitly acknowledge the possibility of type I error due to multiple testing and to emphasize interpretation based on effect sizes, confidence intervals, and consistency across domains rather than statistical significance alone.

We observed that these positive associations were sensitive to model specification. Preeclampsia and resuscitation often occur in the context of other related birth factors (e.g., prematurity and NICU admission), and their estimated effects were not robust to alternative model specifications. When we re-specified the models, these positive associations were no longer present, and estimates were removed from the results.

We have expanded the limitations section to acknowledge that developmental assessment at one year provides an early indicator of neurodevelopment and may not fully capture later-emerging cognitive and behavioral outcomes. **(Page 17, Paragraph 2).**

**References:**

1.	Blakesley RE, Mazumdar S, Dew MA, Houck PR, Tang G, Reynolds CF, 3rd, et al. Comparisons of methods for multiple hypothesis testing in neuropsychological research. Neuropsychology. 2009;23(2):255-64.

2.	Greenland S. Analysis goals, error-cost sensitivity, and analysis hacking: Essential considerations in hypothesis testing and multiple comparisons. 2021;35(1):8-23.

3.	Rothman KJ. No adjustments are needed for multiple comparisons. Epidemiology (Cambridge, Mass). 1990;1(1):43-6.
